# Piezo1-activated mesenchymal stem cells-derived extracellular matrix hydrogel promotes the repair of osteoporotic bone defects through osteogenic and angiogenic coupling

**DOI:** 10.1093/rb/rbag080

**Published:** 2026-05-27

**Authors:** Han Yin, Weiqi Zhang, Dongxuan Wei, Jingguo Chen, Chunxu Fu, Xiaowei Ma, Xuankuai Chen, Xin Xing, Yanbin Zhu, Hongtao Tian, Cao Yang, Yingze Zhang

**Affiliations:** Department of Orthopaedics, Union Hospital, Tongji Medical College, Huazhong University of Science and Technology, Wuhan 430022, China; Department of Orthopaedic Surgery, Hebei Medical University Third Hospital, Shijiazhuang 050051, China; Department of Orthopaedic Surgery, Hebei Medical University Third Hospital, Shijiazhuang 050051, China; Department of Orthopaedics, Union Hospital, Tongji Medical College, Huazhong University of Science and Technology, Wuhan 430022, China; Department of Orthopaedic Surgery, Hebei Medical University Third Hospital, Shijiazhuang 050051, China; Department of Orthopaedic Surgery, Hebei Medical University Third Hospital, Shijiazhuang 050051, China; Department of Orthopaedic Surgery, Hebei Medical University Third Hospital, Shijiazhuang 050051, China; Department of Orthopaedic Surgery, Hebei Medical University Third Hospital, Shijiazhuang 050051, China; Department of Orthopaedic Surgery, Hebei Medical University Third Hospital, Shijiazhuang 050051, China; Department of Orthopaedics, The Second Affiliated Hospital and Yuying Children’s Hospital of Wenzhou Medical University, Wenzhou 325027, China; Department of Orthopaedic Surgery, Hebei Medical University Third Hospital, Shijiazhuang 050051, China; Department of Orthopaedic Surgery, Hebei Medical University Third Hospital, Shijiazhuang 050051, China; Department of Orthopaedics, Union Hospital, Tongji Medical College, Huazhong University of Science and Technology, Wuhan 430022, China; Department of Orthopaedics, Union Hospital, Tongji Medical College, Huazhong University of Science and Technology, Wuhan 430022, China; Department of Orthopaedics, Union Hospital, Tongji Medical College, Huazhong University of Science and Technology, Wuhan 430022, China; Department of Orthopaedic Surgery, Hebei Medical University Third Hospital, Shijiazhuang 050051, China

**Keywords:** Piezo1, Yoda1, cell-derived extracellular matrix, hydrogel, osteogenesis, angiogenesis, osteoporotic bone defect

## Abstract

Osteoporotic bone defects present a significant clinical challenge due to impaired osteogenesis and angiogenesis, often leading to poor healing and bone regeneration. Current therapeutic approaches, including demineralized cancellous bone matrix and synthetic bioactive materials, are often limited by issues such as inadequate donor sources and immune responses. In this study, we developed a novel extracellular matrix hydrogel by optimizing extracellular matrix (ECM) derived from mesenchymal stem cells through preconditioning with Yoda1 (Y-ECM-gel), the Piezo1 mechanosensory channel agonist. The Y-ECM-gel retains the diversity of bioactive factors and structural features found in natural ECM while exhibiting remarkable physical stability. Our *in vitro* experiments demonstrated that Y-ECM-gel significantly promoted the proliferation, migration and osteogenic differentiation of mouse preosteoblast cells (MC3T3-E1), enhancing osteogenesis via activation of the PI3K/AKT signaling pathway. Additionally, Y-ECM-gel promoted the proliferation, migration and angiogenesis of human umbilical vein endothelial cells (HUVECs). *In vivo* experiments, it significantly stimulated bone tissue formation and neovascularization in the osteoporotic bone defect model. These findings indicate that Y-ECM-gel can restore bone homeostasis by promoting osteogenic-angiogenic coupling, providing a promising, minimally invasive and highly effective therapeutic strategy for bone regeneration in osteoporotic conditions.

## Graphical Abstract

**Graphical Abstract:**
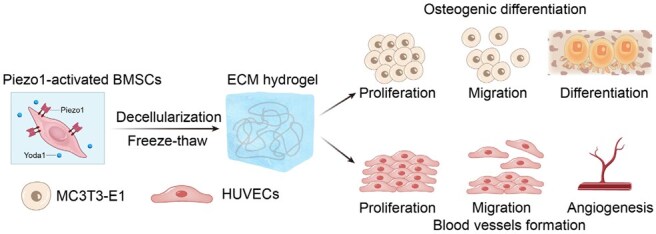

## Introduction

Osteoporosis (OP) is a degenerative disease characterized by reduced bone mass and impaired bone microarchitecture, resulting in increasing bone fragility and fracture risk [1, 2]. With the aging population and changing life-styles, the prevalence of osteoporosis has been increasing annually, leading to a decline in patients’ quality of life and imposing a significant economic burden [3–5]. It has become one of the major global health issues. The bone microstructural deterioration observed in OP patients is typically associated with an imbalance in bone metabolism. This pathological feature is characterized by excessive osteoclast activation and suppressed osteogenic differentiation. This imbalance is caused by multiple factors, including senescence, estrogen deficiency and alterations in mechanical loading [2, 6, 7]. Consequently, osteoporotic bone defects pose a formidable challenge to clinicians, because effective repair requires restoring bone homeostasis and osteogenic differentiation in bone defect regions, while the pathological microenvironment in OP is unfavorable for bone regeneration. Currently, clinical treatment primarily involves a combination of systemic and local therapies. Systemic treatment relies mainly on drug interventions such as bisphosphonates, denosumab and parathyroid hormone. However, oral medications are difficult to effectively concentrate at bone defect regions, and due to their low solubility, they exhibit poor bioavailability and require higher dosages. Furthermore, long-term use may cause systemic side effects and is unable to provide structural support for bone defect regions [8, 9]. Local treatment usually adopts autologous, allogeneic bone transplantation or synthetic bone implant materials, but it is limited by the shortcoming of limited donors, immune rejection and biodegradability which can not fully meet the requirements of effective bone defects treatment [10, 11]. Taken together, an ideal local strategy for osteoporotic bone defects should not only provide structural support but also enhance osteogenic differentiation and improve the regenerative microenvironment in bone defect regions, while avoiding donor limitations and immune rejection. Therefore, it is urgent to develop local bone filling materials with good biodegradability to meet the needs of treatment, which is of great significance for the treatment of osteoporotic bone defects.

Tissue engineering is a promising strategy for treating bone diseases and reconstructing bone defects. With the development of tissue engineering, the limitations of current bone transplantation approaches can be overcome through tissue engineering or regenerative medicine. The extracellular matrix (ECM) is a network structure composed of proteins and glycosaminoglycans, serving as a reservoir for growth factors and other signaling molecules [12]. Decellularized ECM derived from animal tissues has been utilized as a bone-conducting matrix for bone regeneration [13, 14]. However, the application of tissue-derived ECM faces problems such as uncertainty in its composition and structure due to diverse sources, as well as potential immune rejection by the host tissue. Recently, researchers have developed a novel tissue engineering technique involving engineered ECM derived from mesenchymal stem cells (MSCs) [15, 16]. Liu *et al*. [17] found that ECM composite scaffolds derived from osteoblastically differentiated MSCs significantly promoted bone repair by promoting MSCs proliferation and osteogenic differentiation. Compared to natural tissue-derived scaffolds, ECM does not require harvesting from donor tissues, thus, avoiding limitations imposed by tissue availability while eliminating donor site morbidity or infectious disease risks [18]. Furthermore, since ECM is generated through the cell culture process *in vitro*, it allows for quality control and minimizes donor-to-donor variability. Traditional methods for preparing ECM typically involve seeding MSCs into cell culture plates and culturing them in serum-containing medium for 1–2 weeks [19]. However, the ECM generated by this conventional approach often fails to meet the diverse requirements of tissue engineering applications [20]. Consequently, optimizing culture conditions has become a key strategy for enhancing the functionality and performance of the ECM. Common pretreatment strategies include adding specific components to the medium, adjusting serum concentration or altering oxygen levels. However, these approaches often lack specificity and may fail to deliver optimal therapeutic benefits for specific diseases [20]. Therefore, appropriate pretreatment conditions for MSCs are required to enhance the efficacy and bioactivity of the obtained ECM, and establish a solid foundation for constructing MSCs-derived ECM with specific regenerative therapeutic properties.

Mechanical stress is one of the key factors in promoting bone formation and preventing OP through exercise. *In vivo*, osteoblast lineage cells can sense mechanical stresses including tensile, compressive and shear forces, thereby activating intracellular mechanical signaling pathways. Activated cells exhibit enhanced proliferation, migration and osteogenic differentiation capabilities, subsequently participating in bone formation processes to maintain or improve bone mass [21, 22]. Piezo1 is the mechanosensitive channel protein that nonselectively permeates calcium ions (Ca^2+^). It is considered a key sensor for mechanical signals and plays a crucial role in determining bone homeostasis and bone metabolism [23]. It has been demonstrated that Piezo1 promotes osteogenesis and angiogenesis. For instance, osteoblasts lacking Piezo1 exhibit reduced bone formation and decreased bone strength, while administration of Piezo1 agonists increases bone mass [24]. Piezo1 can mediate Ca^2+^ conduction to regulate vascular tension and development by sensing shear force changes induced by blood flow [25]. Bone marrow MSCs (BMSCs) are the multipotent stem cells, and their decreased osteogenic differentiation capacity is one of the key factors contributing to bone homeostasis imbalance [26]. BMSCs can sense mechanical stress stimulation and have been utilized in bone tissue engineering research [27]. Applying mechanical stress activates Piezo1 in BMSCs, which can cause enhanced osteogenic differentiation capacity. Theoretically, transplanting Piezo1-activated BMSCs induced by mechanical stress into bone defect regions could realize bone regeneration. However, limitations in cell transplantation-such as immunogenicity, tumorigenicity and challenges in preservation and transportation-have restricted its further application. Additionally, applying mechanical stress *in vitro* may damage BMSCs, resulting in unstable Piezo1 activation that cannot be effectively controlled. Therefore, alternative activation methods must be explored. Interestingly, Piezo1 does not rely solely on mechanical signal changes. In addition to mechanical stimulation, this channel can also be chemically activated by small molecules such as Yoda1, Jedi1 and Jedi2 [28]. This chemical activation similarly activates Piezo1, permitting Ca^2+^ influx in the absence of mechanical signals and playing a crucial role in osteogenic-angiogenic coupling and bone reconstruction [29]. There were few literatures reporting that Yoda1 activated the Piezo1 channel in BMSCs, and subsequently investigated the effects of the ECM derived from these cells on bone regeneration. Therefore, this study selected Yoda1 to precondition BMSCs, activated their Piezo1 channels, and enhanced the biological functions of the obtained ECM, laying the foundation for promoting bone regeneration.

**Figure 1:**
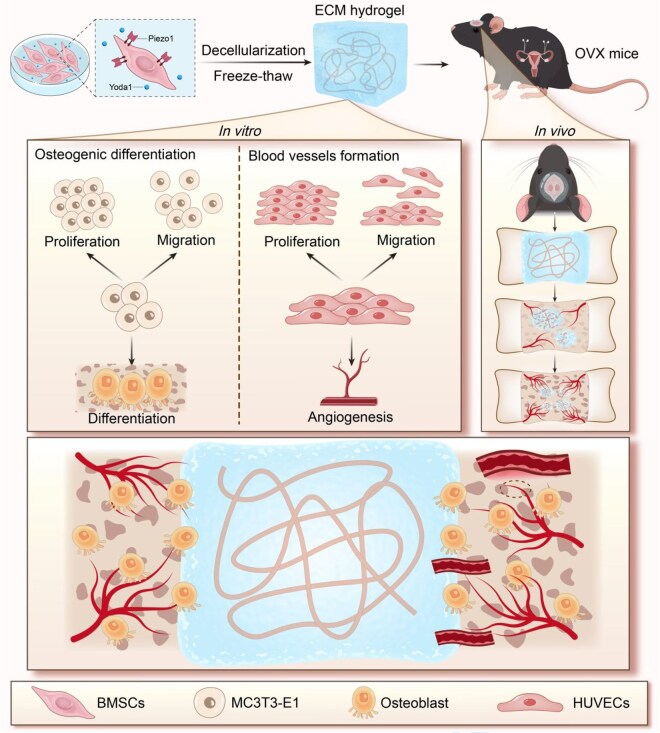
Schematic illustration of the engineered extracellular matrix hydrogel for osteoporotic bone defects therapy.

In summary, we are aiming to develop a MSCs-derived ECM that can both promote osteogenesis and angiogenesis. This goal is achieved through a strategic approach combining Yoda1 pretreatment with *in vitro* culture techniques. We utilized the ability of MSCs to secrete abundant ECM proteins and bioactive molecules during culture and attempted to create the ECM that mimics the dynamic characteristics of natural bone. In particular, we employed Yoda1 to pretreat MSCs, activating the Piezo1 channel to enhance the quality and bioactivity of the generated Yoda1-treated MSCs-derived ECM (Y-ECM). The obtained Y-ECM was then fabricated into the hydrogel (Y-ECM-gel). This Y-ECM-gel exhibits physical stability and hydrogel-like properties, while possessing extremely high-water content and biocompatibility. To our knowledge, this is the first ECM hydrogel composed entirely of ECM components secreted by Piezo1-activated MSCs, without the addition of any crosslinkers or synthetic/natural polymers. The presence of healing-promoting cytokines and growth factors within the Y-ECM-gel provides another significant advantage for tissue regeneration. *In vitro*, the Y-ECM-gel can promote the proliferation, migration and osteogenic differentiation by interacting with preosteoblast cells (MC3T3-E1). It also enhances the proliferation, migration and angiogenesis of human umbilical vein endothelial cells (HUVECs). *In vivo*, this engineered MSCs-derived ECM hydrogel can promote osteogenic-angiogenic coupling in bone defect regions of OP mice and significantly accelerate the bone remodeling process during the early stages of bone defect repair. This study also preliminarily explored the mechanisms underlying its osteogenic effects. These findings will deepen our understanding of how engineered ECM hydrogels promote bone regeneration, and provide a theoretical basis for novel therapeutic approaches to OP bone defects and other OP-related complications. Figure 1 illustrates the technical routes of this study in detail.

## Materials and methods

### Isolation and characterization of BMSCs

Four-week-old C57BL/6J mice (SiPeiFu, China) were euthanized, and femurs and tibias were harvested under aseptic conditions. The bones were flushed with MEM-α to collect bone marrow, which was then centrifuged at 1000 rpm for 5 min. The resulting cell pellet was resuspended in complete MEM-α medium supplemented with 10% fetal bovine serum (FBS) and cultured at cell incubator (37°C, 5% CO_2_). The morphology of primary cells (P0), passage 1 (P1) and P3 BMSCs was observed under the microscope. P3 BMSCs were collected for further analysis, including flow cytometry to identify the expression of surface markers CD31, CD45, CD73, CD90 and CD105. P4 BMSCs were used to assess potential of osteogenesis, adipogenic and chondrogenesis and the differentiation was confirmed by Alizarin Red S (ARS) staining, Oil Red O staining and Alcian Blue staining, respectively. P3-P5 cells were used for subsequent experiments.

### Determination of Yoda1 concentration

#### CCK-8 test

BMSCs were seeded at 2000 cells per well in a 96-well plate. Different concentrations of Yoda1 (0, 0.1 μM, 0.3 μM, 1 μM, 3 μM, 5 μM and 10 μM) (MedChemexpress, USA) were added to the culture medium. On Day 3, cell viability was measured using a CCK-8 kit (Beyotime, China). For detailed steps, please refer to the manufacturer’s instructions.

#### qRT-PCR

After treating BMSCs with different concentrations of Yoda1 for 3 days, total RNA was extracted and reverse transcribed into cDNA. Quantitative real-time polymerase chain reaction (qRT-PCR) analysis was performed to measure the expression of Piezo1, with GAPDH as an internal control. The primer sequences are listed in Supplementary Table S1.

#### Western blot experiment

RIPA lysis buffer (Beyotime, China) containing 1 mM phenylmethanesulfonyl fluoride (PMSF) was used to extract the protein from cultured cells. The amount of protein was measured by the BCA Protein Assay kit (Beyotime, China). The proteins were separated in an SDS-PAGE gel, and then, transferred to a PVDF membrane (0.45 μm, Merck Millipore, USA), blocked with 5% skimmed milk (Servicebio, China) for 2 h, treated with primary antibodies at 4°C for an overnight period, and then, incubated with a secondary goat anti-rabbit antibody that had been HRP-conjugated for 1 h at room temperature. The enhanced chemiluminescence (ECL) substrate kit (Servicebio, China) was then used to identify the proteins, and Image J was used to quantify their expression. The expression was compared to the internal reference value (GAPDH).

### BMSCs decellularization

BMSCs were seeded at a density of 2 × 10^4^ cells/cm^2^ on tissue culture plates (TCP) and cultured for 24 h. The culture medium was replaced with 10% FBS MEM-α medium containing 3 μM Yoda1, and the cells were cultured for 9–10 days, with medium containing 3 μM Yoda1 changes every 3 days. BMSCs cultured without Yoda1 served as the control. To obtain decellularized BMSCs-derived ECM, cells were washed twice with PBS and treated with decellularization solution (0.5% Triton-X 100 and 20 mM NH_4_OH) at 37°C for 5 min. After removing the decellularization solution, 100 U/mL DNase I was added, and the mixture was incubated at 37°C for 2 h. The ECM was washed several times with PBS, and the decellularization process was monitored microscopically. ECM obtained from control and Yoda1-treated BMSCs was named ECM and Y-ECM, respectively, and stored at 4°C for future use.

### Assessment of decellularization efficiency

#### DNA and glycosaminoglycans quantification

Residual DNA content before and after decellularization was measured using a commercial DNA extraction kit (TIANGEN, China), and DNA concentration was quantified spectrophotometrically to evaluate decellularization efficiency. Glycosaminoglycans (GAGs) content in the ECM were assessed using an enzyme-linked immunosorbent assay (ELISA) kit (TIANGEN, China).

#### Immunofluorescence staining

Samples before and after decellularization were fixed in 4% paraformaldehyde for 15 min and washed three times with PBS. After blocking with blocking solution at room temperature for 1 h, samples were incubated with the primary antibody against fibronectin (Bioss, China) overnight at 4°C, followed by incubation with the secondary antibody at room temperature for 1 h. Nucleus was counterstained with DAPI. Images were captured using the fluorescence microscope.

### Preparation of ECM hydrogels

To fabricate ECM hydrogels (ECM-gel and Y-ECM-gel), confluent BMSCs cultured on 100 mm plates were decellularized and the ECM was gently scraped off and transferred to 50 mL conical tubes containing deionized water. The ECM suspension was centrifuged at 3500 rpm for 8 min, and the pellet was frozen overnight at −80°C, followed by thawing at 37°C to induce physical crosslinking and gelation. The resulting ECM hydrogels were carefully removed with sterile forceps and stored at −20°C for further use.

### Characterization of ECM hydrogels

#### The rheological property test

The rheological property of the hydrogels was assessed at 37°C under 5% shear strain. During this process, the storage modulus (*G′*) and loss modulus (*G*″) were measured.

#### SEM and FTIR

The microstructure of ECM, ECM-gel, Y-ECM and Y-ECM-gel was examined after freeze-drying overnight. Samples were imaged using scanning electron microscopy (SEM) to analyze surface ultrastructure. The distribution of elements was imaged by Energy Dispersive X-ray Spectroscopy (EDS). In addition, Fourier transform infrared spectroscopy (FTIR) spectrophotometer was used to evaluate molecular composition. All the FTIR spectra were acquired in the 1000–4000 cm^−1^ range at a resolution of 4.0 cm^−1^ with 16 scans per sample.

#### Immunofluorescence staining

Expression of fibronectin within ECM-gel and Y-ECM-gel was examined using immunofluorescence (IF) staining. Samples were imaged with the confocal laser scanning microscope (CLSM). Detailed staining procedures followed those described in the earlier section.

#### Enzyme-linked immunosorbent assay

According to the ELISA kit instructions, determine the concentrations of bone morphogenetic protein 2 (BMP2), vascular endothelial growth factor (VEGF) or transforming growth factor-β (TGF-β) in Y-ECM-gel and ECM-gel. Set up standard and sample wells, add 50 μL of color developer and incubate at 37°C in the dark. After 15 min, add stop solution to terminate the reaction. Measure the absorbance (450 nm) after 15 min.

#### Proteomic analysis

The protein lysates of ECM-gel and Y-ECM-gel were prepared and analyzed by LC-MS/MS using the Orbitrap Exploris 240 mass spectrometer (USA). Raw data were analyzed using MaxQuant 1.5.3.30 software and the Andromeda search engine (Cox & Mann, 2008). By comparing peptides and proteins with the Uniprot database. In peptide spectrum matching (PSM), the false discovery rate (FDR) was set to 1%, and only peptides with FDR below 1% were selected for identification. For protein quantification, the label-free quantification of identified peptides was applied to protein annotations. Unique and razor peptides were selected as quantitative peptides, and protein abundance was calculated by summing the intensities of all quantitative peptides. Abundance was normalized to the total peptide quantity and scaled with the average across all samples set to 100. The default parameters of MaxQuant were used for protein quantification. Differentially expressed proteins (DEPs) were filtered with the threshold of fold change greater than 2 and *P* value less than 0.05. The selected DEPs were included in further functional analysis based on Gene Ontology (GO) and Kyoto Encyclopedia of Genes and Genomes (KEGG) databases.

### *In vitro* biocompatibility assessment

#### Preparation of extract liquid

The hydrogels were immersed in 75% ethanol for 30 min, followed by three washes with PBS. It was, then, exposed to ultraviolet (UV) light for 1 h. Finally, the hydrogels were immersed in 3 mL simple culture medium and incubated in a cell culture incubator for 24 h to obtain the extract liquid.

#### Cell adhesion

MC3T3-E1 and HUVECs were seeded onto ECM-gel and Y-ECM-gel separately. After two days of co-culture, cell nuclei were counterstained with DAPI and observed under the CLSM.

#### Cell morphology

ECM-gel and Y-ECM-gel were placed in the upper chamber of the transwell system and cultured with complete medium. MC3T3-E1 and HUVECs were seeded in the lower chamber at a density of 3000 cells per well. After 3 days, cells were fixed, permeabilized with 0.5% Triton-X and stained with phalloidin to label F-actin. Detailed staining protocols followed manufacturer instructions.

#### Cell viability

The viability of MC3T3-E1 and HUVECs was assessed using the Calcein-AM/PI staining kit (Beyotime, China). Viable cells were visualized and quantified under the microscope. Detailed staining procedures followed the manufacturer’s instructions.

By staining MC3T3-E1 and HUVECs with Calcein-AM/PI (Beyotime, China), we assessed cell viability after 3 days of co-culture. The co-culture system was established using the previously described method. Specific staining procedures can be found in the manufacturer’s instructions.

### Cell proliferation assay

#### CCK-8 test

The co-culture system was established using the methods described above. After co-culture of 1, 3 and 5 days, CCK-8 assays were performed to evaluate the effects of the two hydrogels on cell proliferation. Absorbance values (OD values) were measured at 450 nm using the microplate reader. Refer to the manufacturer’s instructions for detailed procedures.

#### EdU assay

The co-culture system was established using the methods described above. After co-culture of 3 and 5 days, proliferation was further assessed using the EdU assay to visualize proliferating cells by fluorescence staining. Detailed protocols followed manufacturer’s instructions.

#### Cell cycle assay

The co-culture system was established using the methods described above. Cells cultured in complete medium were set as the control group. After co-culture of 3 days, cell cycle was assessed with the cell cycle analysis kit (Beyotime, China). Detailed protocols followed manufacturer’s instructions.

### *In vitro* osteogenic and angiogenic capabilities

#### Scratch assay of MC3T3-E1 and HUVECs

MC3T3-E1 and HUVECs were seeded at a density of 3 × 10^5^ cells per well in a 6-well plate. Two straight lines were scratched in each well using a sterile 200 μL pipette tip. After washing with PBS to remove detached cells, ECM-gel and Y-ECM-gel were added. Migration was observed at 0, 12 and 24 h under the microscope. The wound closure area was quantified using ImageJ software.

#### Transwell assay of MC3T3-E1 and HUVECs

ECM-gel and Y-ECM-gel were placed in the lower chamber of the Transwell plate. MC3T3-E1 and HUVECs were seeded in the upper chamber at a density of 2 × 10^4^ cells per well. After 24 h incubation, nonmigrated cells were gently removed from the upper chamber, and migrated cells were fixed in 4% paraformaldehyde for 15 min. The cells were then stained with 0.1% crystal violet for 10 min, and images of the migrated cells were captured under the microscope. The number of migrated cells were quantified using ImageJ software.

#### Osteogenesis evaluation of MC3T3-E1

MC3T3-E1 cells were seeded in the lower chamber of the Transwell plate at a density of 3 × 10^4^ cells per well. When the cells reached 60% confluence, the medium was replaced with osteogenic differentiation medium (OriCell, China). ECM-gel and Y-ECM-gel were placed in the upper chamber. The control group was cultured without any hydrogels. After 7 days, alkaline phosphatase (ALP) activity was measured using an ALP assay kit (Beyotime, China). After 21 days, mineralized nodules were stained with ARS, and calcium deposition was quantified using the ARS quantification kit (Solarbio, China).

#### RNA sequencing analysis

For RNA sequencing (RNA-seq) analysis, MC3T3-E1 were co-cultured with ECM-gel and Y-ECM-gel for 3 days, respectively. After 3 days of co-culture, cells were harvested and performed RNA-seq using next-generation sequencing technology on the Illumina NovaSeq 6000 platform. Total RNA was obtained using the RNeasy Micro Kit and then purified with the RNA Clean XP Kit and RNase-Free DNase Set. After completing library construction, RNA is fragmented and reused for cDNA synthesis. All libraries were quantified using the Qubit^®^ 2.0 Fluorometer and Agilent 4200 System. After determining fold changes and statistical significance, differentially expressed genes (DEGs) with fold changes > 2 or < 0.5 and *P* values < 0.05 were considered statistically significant.

#### Tube formation assay of HUVECs

To investigate the effects of two hydrogels on HUVECs angiogenesis, we conducted tubule formation experiments *in vitro*. In brief, HUVECs were preincubated overnight with two hydrogels before being seeded at a density of 2 × 10^4^ cells per well into 96-well plates pretreated with matrix gel. After 6 h incubation, cells were observed under the optical microscope and photographed. The number of junctions and total branching length were quantified using Image J software.

#### qRT-PCR

MC3T3-E1 and HUVECs were collected separately after 7 days of two hydrogels treatment. Total RNA was extracted and reverse transcribed into cDNA. According to the manufacturer’s instructions, the expression levels of osteogenesis-related genes (ALP, BMP2, OCN, Runx2 and Col 1) and angiogenesis-related genes (CD31, VEGF, HIF-1α and bFGF) were detected using qRT-PCR analysis, where the Ct value was standardized to GAPDH in the same sample. The primer sequences are shown in Supplementary Table S1.

#### IF staining

MC3T3-E1 and HUVECs were collected separately after 7 days of two hydrogels treatment. The expression levels of BMP2 (Bioss, China) and osteocalcin (OCN) (Proteintech, China) in MC3T3-E1 were detected using IF staining. Detect the expression levels of CD31 (Proteintech, China) and CD34 (Bioss, China) in HUVECs. For more detailed steps on IF staining, refer to the earlier section.

### *In vivo* new bone formation evaluation

#### Establishment of ovariectomized model

Twenty C57BL/6J mice (female, 20–25 g) were purchased from Beijing SiPeiFu operation. After one week of adaptive feeding, the mice were used for experiments. All animal experiments were approved by the Animal Ethics Committee of the Hebei Medical University Third Hospital (Permit number 2024-019). All procedures were conducted in the SPF environment. First, mice were anesthetized with 5% isoflurane, placed in prone position and hair shaved along both sides of the dorsal spine. The surgical site was disinfected with iodine solution. A longitudinal incision of approximately 0.5 cm was made through the skin and muscle. Carefully exposed the pink ovaries, ligated the uterine-ovarian junction and excised the ovaries. Muscle and skin were then sutured layer by layer, and the animals were returned to their cages. All animals were maintained for 2 months postsurgery to allow osteoporosis development. Three mice were randomly selected and euthanized to validate the success of the ovariectomized (OVX) model.

#### Establishment of cranial defect model

OVX mice were anesthetized with 5% isoflurane and secured to the surgical table. The cranial region was exposed by incising the skin and soft tissue with the scalpel. After exposing the bone surface, a standardized circular bone defect (4 mm diameter, 2 mm depth) was created in the skull using a dental burr. Three groups were randomly divided according to Figure 9A. After implanting the two hydrogels, the incision was closed using 4-0 absorbable sutures. Postoperatively, the mice were placed in a warm, dry environment. Simultaneously, 10 × 10^4^ U penicillin were administered via intramuscular injection for 3 days to prevent infection. The recovery of the mice should be closely monitored, and necessary care and housing conditions should be provided. After 8 weeks postsurgery, all mice were euthanized by CO_2_ inhalation in accordance with the rules of the animal ethics committee.

#### Micro-CT

Micro-CT was used to assess cranial bone regeneration in mice. CT software was employed to visualize and perform three-dimensional (3D) reconstruction of the results, and analyze bone mineral density (BMD), trabecular thickness (Tb.Th), bone volume fraction (BV/TV) and trabecular separation (Tb.Sp).

#### Histological analysis and IF staining

Following Micro-CT, specimens were fixed in 4% paraformaldehyde for 48 h, and then, decalcified, embedded and sectioned (5 μm thick, sagittal orientation). Observation of cranial bone regeneration in each group was performed using Hematoxylin and eosin (H&E) (Solarbio, China), Masson’s trichrome staining (Solarbio, China) and IF staining (OCN: Proteintech, China; Runx2: Proteintech, China). Vascular regeneration was assessed via IF staining of vascular markers (CD31: Proteintech, China; EMCN: Proteintech, China).

### Statistical analysis

The data are expressed as the mean ± standard deviation (SD) and analyzed using GraphPad Prism 10.0 software. All experiments were repeated at least three times independently. Significance testing was performed using Student’s *t*-test and one-way analysis of variance (ANOVA). *P* values < 0.05 indicate statistically significant differences.

## Results and discussion

### Confirmation of Yoda1 optimal concentration

Due to variations in experimental conditions and cell status, the selection of Yoda1 concentrations presents diversity [30, 31]. The optimal Yoda1 concentration maintains cell viability while maximally activating the Piezo1 channel. We examined the optimal concentration of Yoda1 for culturing BMSCs *in vitro*. First, we characterized the extracted BMSCs. We observed the morphological characteristics of BMSCs. Results showed that P0 BMSCs exhibited irregular shapes and gradually elongated after adhesion. After passaging, P1 and P3 cells gradually became shuttle-shaped and more uniform and showed typical stem cell-like morphology (Supplementary Figure S1A). Subsequently, their multidirectional differentiation potential was assessed. ARS staining revealed that the cells could form calcified nodules, Oil Red O staining demonstrated the presence of abundant lipid droplets and Alcian Blue staining indicated the cells possessed chondrogenic secretion capacity. These results confirmed that the extracted BMSCs possessed osteogenic, adipogenic and chondrogenic differentiation potential (Supplementary Figure S1B). It was further demonstrated by flow cytometry analysis of cell surface markers (Supplementary Figure S1C) that these cells negatively expressed hematopoietic and endothelial cell markers CD31 (0.31%) and CD45 (0.30%), while highly expressing MSCs-specific markers CD73 (89.5%), CD90 (99.6%) and CD105 (95.4%), consistent with the phenotypic characteristics of BMSCs. These results indicated that we successfully isolated BMSCs.

Next, we evaluated the effects of different Yoda1 concentrations on BMSCs viability using the CCK-8 assay. Results demonstrated a distinct biphasic effect of different Yoda1 concentrations on BMSCs viability. Compared to the control group (0 μM), low-concentration Yoda1 treatment (0.1, 1 and 3 μM) did not inhibit BMSCs viability. However, at higher concentrations (5 and 10 μM), BMSCs viability decreased significantly, indicating that high doses of Yoda1 exerted an inhibitory effect on BMSCs viability (Supplementary Figure S2A).

The effective activation of Piezo1 in BMSCs was the theoretical basis for our experiments. Finally, we further determined the concentration of Yoda1 by detecting its expression in BMSCs via qRT-PCR. Results showed that the relative expression level of Piezo1 gradually increased with increasing Yoda1 concentration, exhibiting a significant dose-dependent upregulation trend. The highest expression level of Piezo1 was observed at 3 μM Yoda1 (Supplementary Figure S2B). Western blot (WB) analysis was performed to evaluate the effect of Yoda1, a selective Piezo1 agonist, on the protein levels of Piezo1. As shown in Supplementary Figure S2C, the protein expression of Piezo1 exhibited a significant, dose-dependent increase following treatment with varying concentrations of Yoda1 (0, 0.1, 0.3, 1 and 3 μM). Quantitative analysis (Supplementary Figure S2D) further confirmed that Yoda1 treatment significantly enhanced Piezo1 expression. These results demonstrate that Yoda1 effectively induces the expression of Piezo1 protein in a dose-dependent manner. Based on the above results, 3 μM Yoda1 was selected for subsequent experiments.

### Preparation and characterization of Y-ECM-gel

**Figure 2:**
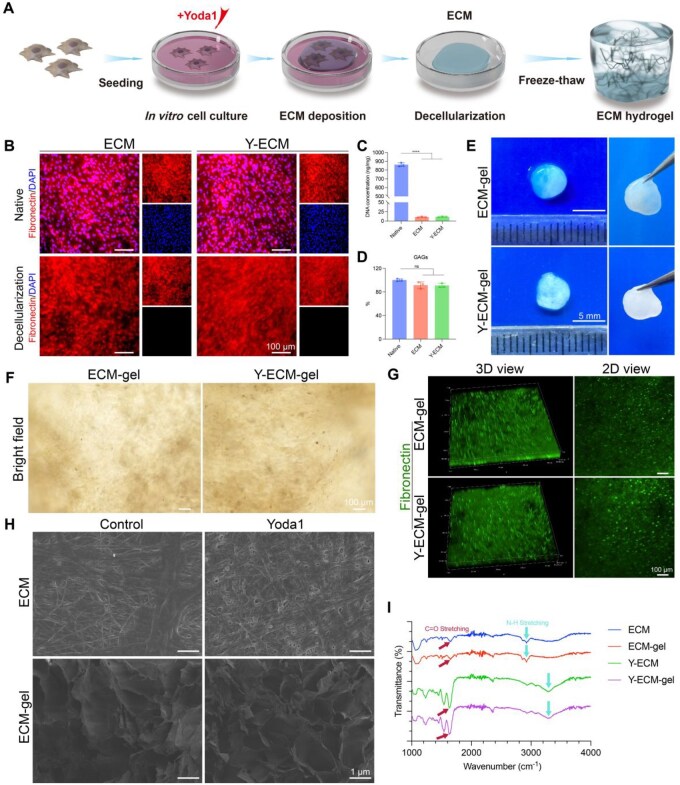
Preparation and characterization of Y-ECM-gel. (**A**) Fabrication process of Y-ECM-gel. (**B**) IF staining of fibronectin before and after decellularization. (**C**) DNA content in ECM before and after decellularization (*n* = 3). (**D**) GAGs content in ECM before and after decellularization (*n* = 3). (**E**) Appearance of ECM-gel and Y-ECM-gel and these gripped by forceps. (**F**) Microscopic view of ECM-gel and Y-ECM-gel. (**G**) IF staining of fibronectin in ECM-gel and Y-ECM-gel. (**H**) Surface texture of ECM (2D), ECM-gel (3D), Y-ECM (2D) and Y-ECM-gel (3D) as observed via SEM. (**I**) Comparison of the functional groups between ECM, ECM-gel, Y-ECM and Y-ECM-gel as assessed via FTIR. Error bars denote means ± SD, ****P < 0.0001, ns indicates no significance.

Decellularization technology has been proved to be an effective method to prepare bioactive ECM materials [32, 33]. This technology can completely remove cellular components while preserving the protein structure and biochemical properties of the ECM, thereby maintaining its essential intrinsic signaling required for cell recruitment and tissue regeneration [15, 34]. To assess whether activation of the mechanosensory channel Piezo1 in BMSCs affects ECM function, we incubated BMSCs for 10 days with Yoda1. Following mild decellularization, the ECM was harvested, centrifuged and subjected to freeze-thaw cycles. Following the above procedures, we successfully synthesized the bioactive engineered ECM hydrogel (Y-ECM-gel; Figure 2A). The ECM hydrogel (ECM-gel) prepared from BMSCs without Yoda1 treatment served as the control group. Microscopic observations revealed that before decellularization, BMSCs showed stratified growth in a vortex-like or track-like pattern. After decellularization, both decellularized ECM had lost the morphology of BMSCs and exhibited fibrous network structures in the surface (Supplementary Figure S3). IF staining revealed that, with or without Yoda1 treatment, fibronectin exhibited an ordered fibrous structure before decellularization, and the nuclear architecture was still intact. After decellularization, intact cellular structures including nuclei were disrupted, while ECM components were well preserved (Figure 2B). Regarding decellularization efficiency, DNA content decreased significantly from 862.13 ± 21.48 ng/mg in native BMSCs to less than 20 ng/mg after decellularization (ECM: 10.80 ± 1.50 ng/mg; Y-ECM: 11.47 ± 1.24 ng/mg), which was far below the 50 ng/mg threshold (Figure 2C). In contrast, GAGs content remained stable over the whole process (Figure 2D). The above results indicate that our preparation method effectively preserves key extracellular matrix components, including collagen and GAGs. Next, we employed the freeze-thaw method to aggregate ECM components densely, and cross-link a large number of ECM molecules through physical processes. This triggered protein-protein interactions, and eventually formed a pure ECM hydrogel (ECM-gel and Y-ECM-gel). Macroscopically, both ECM hydrogels exhibited a translucent white gel-like appearance, displaying the fundamental characteristics of classical hydrogels (Figure 2E). They also retained exceptionally high-water content, which was confirmed by measuring the difference between wet and dry weights (Supplementary Figure S4). Y-ECM-gel exhibits viscoelastic behavior. The mechanical properties of Y-ECM-gel, as evaluated by the G’ and G”, are superior to those of ECM-gel (Supplementary Figure S5). Optical microscopy images reveal interconnected fiber networks within both hydrogels (Figure 2F). IF results further revealed that both hydrogels retained the major component of the ECM-fibronectin (green) (Figure 2G). Compared to the ECM-gel, the Y-ECM-gel exhibited a more numerous and complex fibrillar network structure. SEM revealed that after decellularization, the cellular architecture of BMSCs cultured on the plates had completely disappeared, exposing a stacked fibrous network structure regardless of Yoda1 treatment. These images clearly demonstrated that the fibrous network structure of Y-ECM-gel exhibited a more uniform thickness compared to ECM-gel (Figure 2H, Upper). After freeze-drying, both hydrogels exhibited a loose, porous structure and the pore size distribution of Y-ECM-gel was more uniform than ECM-gel (Figure 2H, Bottom). The element mapping scanning results exhibited that Y-ECM-gel and ECM-gel were enriched by carbon (C) and oxygen (O) and contained a small amount of phosphorus nitrogen (N) and sulfur (S). However, after treatment with Yoda1, the contents of S increased and the element distribution was more uniform (Supplementary Figure S6). Since ECM-gel and Y-ECM-gel (3D) are prepared from ECM and Y-ECM (2D), FTIR confirmed that ECM and ECM-gel share identical functional groups, which was verified by protein characteristic peaks such as the C = O (1600–1800 cm^−1^) and N-H (2800–3000 cm^−1^; Figure 2I). Y-ECM-gel and ECM-gel exhibit differences in N-H stretching vibrations, which demonstrates that Yoda1 treatment has influenced the chemical composition of the hydrogel.

### Proteomic analysis

Yoda1 treatment has been shown to modulate the secretion of ECM components in MSCs, including collagen and fibronectin, which can influence ECM composition and structure [35]. This remodeling of the ECM may affect MSCs behavior, such as adhesion, migration and differentiation, as ECM stiffness and composition are key regulators of stem cell fate [36]. We hypothesize that Yoda1 activation enhances ECM deposition and promotes tissue regeneration by altering the MSCs secretome and ECM architecture. Studies should investigate the exact mechanisms through which Yoda1-induced ECM changes impact MSCs functions.

**Figure 3:**
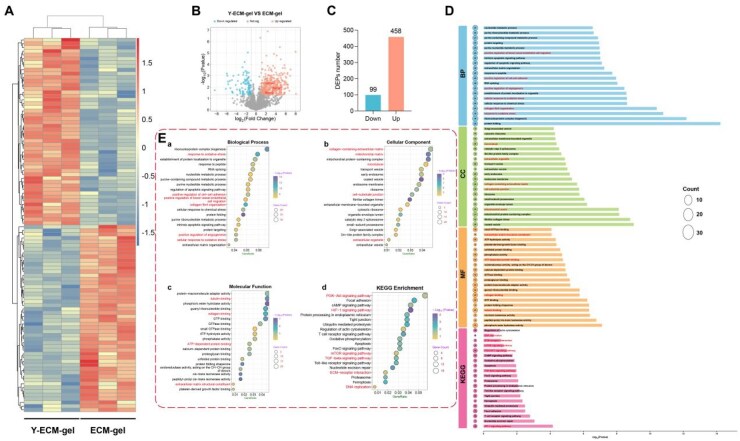
Proteomic analysis of Y-ECM-gel. (**A**) Heatmap analysis indicated 50 DEPs in the two groups. (**B**) Volcano plot showing significantly upregulated (orange dots) and downregulated (turquoise dots) proteins in Y-ECM-gel, compared to ECM-gel. (**C**) Numbers of DEPs in the two groups. (**D**) GO analysis of DEPs in Y-ECM-gel, categorized into BP, CC and MF. (**E_a-c_**) GO enrichment analysis of DEPs in Y-ECM-gel. The top 20 enriched terms of the three categories in (**D**) were, respectively, presented as bubble charts. (**E_d_**) KEGG pathway analysis of DEPs in Y-ECM-gel.

To better understand the properties of Y-ECM-gel and comprehensively characterize how Piezo1 activation influences ECM composition, we extracted proteins of ECM-gel and Y-ECM-gel and performed LC-MS/MS analysis. A total of 4643 proteins were identified (Figure 3B), among which 557 were DEPs. Specifically, 458 were significantly upregulated in Y-ECM-gel, while 99 were significantly downregulated. Importantly, when analyzing proteins associated with osteogenesis promotion, we found that Piezo1 activation significantly modulated the expression of Col1a1, Col1a2 and Runx2 (Figure 3C). The heatmap displays 50 upregulated and 50 downregulated proteins in Y-ECM-gel (Figure 3A). We then focused on these DEPs and performed functional analysis. Regarding functional annotation based on the GO database, the DEPs were annotated across multiple terms within three domains: “Biological Process (BP),” “Cellular Component (CC),” and “Molecular Function (MF),” indicating the presence of multiple functional proteins in the Y-ECM-gel (Figure 3D). For BP (Figure 3Ea), enrichment analysis revealed that DEPs were involved in response to oxidative stress, positive regulation of cell–cell adhesion, positive regulation of blood vessel endothelial cell migration, collagen fibril organization, positive regulation of angiogenesis and cellular response to oxidative stress, etc. For CC (Figure 3Eb), DEPs were located in collagen-containing extracellular matrix, mitochondrial matrix, microtubule, cell-substrate junction and extracellular organelle, etc. For MF (Figure 3Ec), DEPs primarily perform functions such as tubulin binding, collagen binding, ATP-dependent protein binding and extracellular matrix structural constituent. Furthermore, we explored the pathways involved in DEPs through KEGG pathway analysis. Results indicated that these DEPs were associated with the PI3K/AKT signaling pathway, HIF-1 signaling pathway, mTOR signaling pathway, TGF-beta signaling pathway, ECM-receptor interaction and DNA replication (Figure 3Ed). These pathways play crucial roles in bone regeneration and angiogenesis. Overall, our Y-ECM-gel is a complex mixture of bioactive molecules, each with a specific function, but which are equally important in bone regeneration. These findings highlight the tremendous potential of Y-ECM-gel in supporting bone homeostasis and enhancing tissue regeneration.

### *In vitro* biocompatibility evaluation

**Figure 4:**
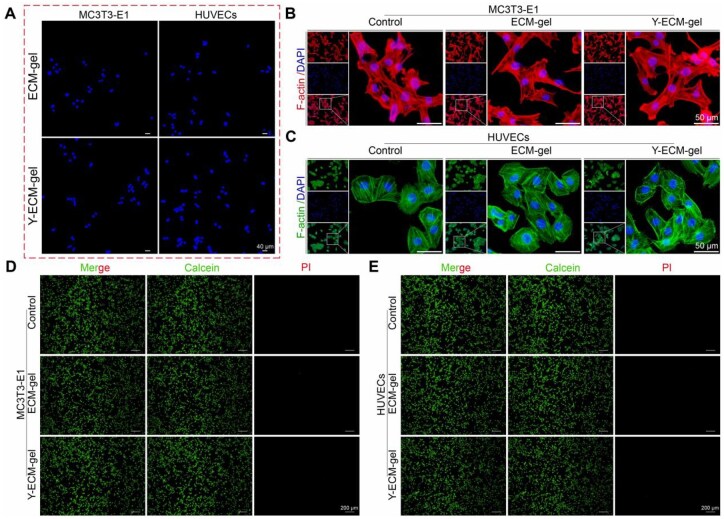
*In vitro* biocompatibility evaluation. (**A**) Nuclei distribution of MC3T3-E1 and HUVECs on the ECM-gel or Y-ECM-gel surface. (**B**) IF staining of F-actin in MC3T3-E1 cultured with ECM-gel or Y-ECM-gel for 3 days. (**C**) IF staining of F-actin in HUVECs cultured with ECM-gel or Y-ECM-gel for 3 days. (**D**) Live/dead assay of MC3T3-E1 cultured with ECM-gel or Y-ECM-gel for 3 days. (**E**) Live/dead assay of HUVECs cultured with ECM-gel or Y-ECM-gel for 3 days. Live cells appear green, and dead cells appear red.

Excellent cytocompatibility is a fundamental prerequisite for the application of hydrogel materials [37–39]. In cell adhesion assays, MC3T3-E1 and HUVECs were able to adhere to both ECM-gel and Y-ECM-gel (Figure 4A). MC3T3-E1 and HUVECs were collected after co-culture with the two hydrogels, and their morphological characteristics were observed through cytoskeletal staining. As shown in Figure 4B and C, MC3T3-E1 and HUVECs fully extended and formed pseudopodia. Furthermore, live/dead staining was employed to assess the cytotoxicity of both hydrogels. As shown in Figure 4D and E, only a very few dead cells (red) were observed in all groups by Day 3 and the majority of cells remained viable (green), indicating that both hydrogels exhibit cell affinity. These results demonstrate that Y-ECM-gel and ECM-gel exhibit excellent biocompatibility and function as effective cell adhesion supports, which provide a robust three-dimensional (3D) environment capable of supporting endogenous cell growth, and provide theoretical support for future clinical applications.

### Y-ECM-gel promotes MC3T3-E1 and HUVECs proliferation *in vitro*

**Figure 5:**
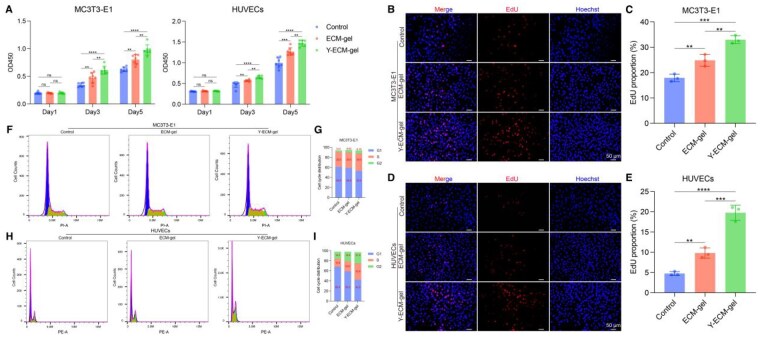
Y-ECM-gel promotes MC3T3-E1 and HUVECs proliferation *in vitro*. (**A**) CCK-8 results of MC3T3-E1 and HUVECs proliferation under the treatment of ECM-gel or Y-ECM-gel (*n* = 7). (**B**, **D**) EdU staining results of MC3T3-E1 or HUVECs (EdU: red, nucleus: blue) (*n* = 3). (**C**, **E**) Semi-quantitative analysis of EdU-positive MC3T3-E1 or HUVECs ratio. (**F**, **H**) Flow cytometry results of the cell cycle of MC3T3-E1 and HUVECs treated with ECM-gel or Y-ECM-gel for 3 days. (**G**, **I**) The proportion of G1, S and G2 in MC3T3-E1 or HUVECs. Error bars denote means ± SD, **P < 0.01, ***P < 0.001, ****P < 0.0001, ns indicates no significance.

Maintaining a good status of cell proliferation is one of the effective factors influencing bone regeneration. The effect of Y-ECM-gel on cell proliferation was evaluated using the CCK-8 assay. Quantitative analysis results indicate (Figure 5A) that the proliferation capacity of MC3T3-E1 and HUVECs increased gradually with increasing co-culture times. After 1 day of co-culture, there was no statistically significant difference in the proliferation capacity of MC3T3-E1 and HUVECs among the three groups. After 3 and 5 days, the proliferation capacity in the ECM-gel group and Y-ECM-gel group was significantly higher than the control group, whereas the Y-ECM-gel group exhibited greater proliferation than the ECM-gel group. EdU assay results demonstrated that the number of EdU-positive cells in both the ECM-gel group and Y-ECM-gel group was significantly higher than the control group, and that the Y-ECM-gel group exhibited the highest number of EdU-positive cells (Figure 5B and D). Quantitative analysis results also revealed the same increasing trend (Figure 5C and E). To investigate the mechanism by which Y-ECM-gel promotes cell proliferation, we performed cell cycle analysis on MC3T3-E1 and HUVECs under different treatments. Results showed that in both the ECM-gel group and Y-ECM-gel group, the proportion of cells in S and G2 phases was significantly higher than in the control group for both MC3T3-E1 (Figure 5F and G) and HUVECs (Figure 5H and I), and the highest proportion was observed in the Y-ECM-gel group. Meanwhile, the percentage of cells in the G1 phase decreased, with the lowest proportion observed in the Y-ECM-gel group. This suggests that Y-ECM-gel may promote the proliferation of MC3T3-E1 and HUVECs by regulating cell cycle activity. The proliferation-promoting capacity of Y-ECM-gel may be attributed to Yoda1 which activates the Piezo1 channel, which then affects the ECM secreted by cells. The ECM secreted contains a richer concentration of growth factors and a more favorable microenvironment, thus, promoting cell proliferation. The above experiments demonstrated the positive regulatory effect of Y-ECM-gel on cell proliferation through three distinct methods. These findings provide a guidance basis for our subsequent experiments.

### Y-ECM-gel promotes MC3T3-E1 osteogenesis *in vitro*

**Figure 6:**
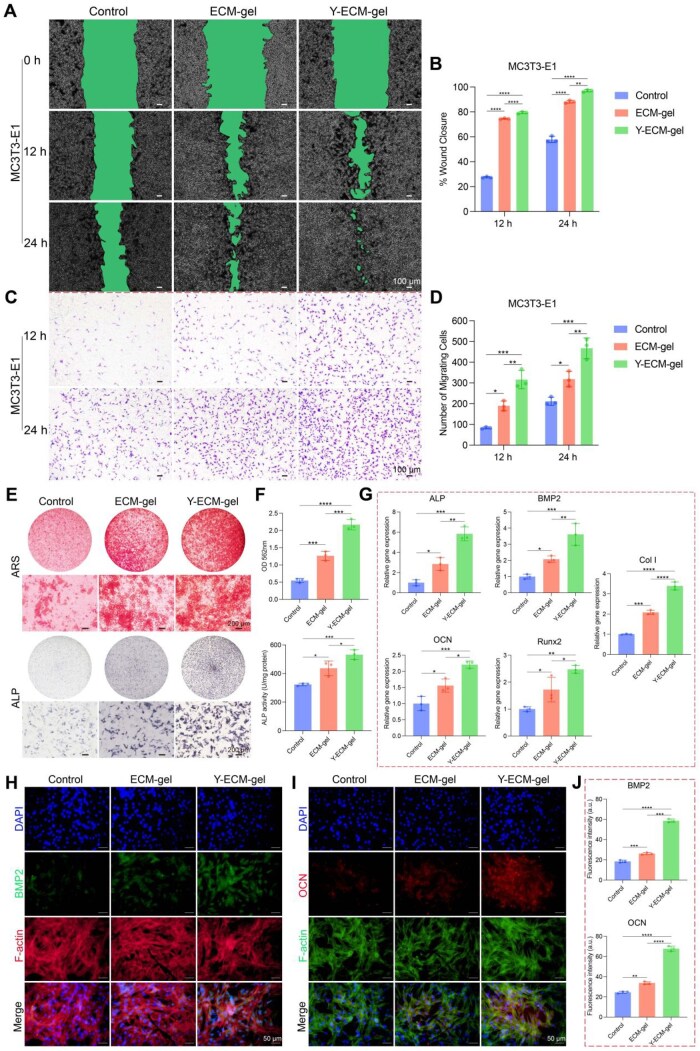
Y-ECM-gel promotes MC3T3-E1 osteogenesis *in vitro*. (**A**) Scratch assay verified the effect of Y-ECM-gel on promoting MC3T3-E1 migration at 0, 12 and 24 h. (**B**) Quantitative analysis of the wound closure (*n* = 3). (**C**) Transwell assay accessing the migration activity of MC3T3-E1 cultured with the Y-ECM-gel where the migrated cells were stained with crystal violet. (**D**) Quantitative analysis of the number of migrated MC3T3-E1 (*n* = 3). (**E**) Representative images of ALP staining and ARS staining. (**F**) Quantitative analysis of calcium deposition and ALP activity (*n* = 3). (**G**) The osteogenesis-related genes (ALP, BMP2, OCN, Runx2 and Col I) expression of MC3T3-E1 on Day 7, respectively (*n* = 3). (**H**, **I**) Representative images of BMP2 and OCN IF staining of MC3T3-E1. (**J**) Semi-quantitative analysis of fluorescence intensity (*n* = 3). Error bars denote means ± SD, *P < 0.05, **P < 0.01, ***P < 0.001, ****P < 0.0001.

The osteogenesis capacity of cells is crucial for *in vivo* bone defect regeneration and repair, while the recruitment of a greater number of cells to the defect region is also essential. First, to determine the recruitment capacity of our Y-ECM-gel, we employed a scratch assay. MC3T3-E1 cells were seeded in the lower chamber, with the Y-ECM-gel placed in the upper chamber. The release of bioactive molecules from the Y-ECM-gel promoted cell migration toward the scratch area. Interestingly, compared to the control group and ECM-gel group, the Y-ECM-gel group demonstrated significantly improved wound closure at both 12 h and 24 h, indicating its superior capacity to promote cell migration (Figure 6A). Statistical analysis also revealed significant differences (Figure 6B). Additionally, we employed Transwell chemotaxis assays to further investigate the effects of Y-ECM-gel on the migration behavior of MC3T3-E1. In this experiment, the Y-ECM-gel is placed in the lower chamber, while MC3T3-E1 are added to the upper chamber. Research findings indicate that the number of cells migrating to the lower chamber containing ECM-gel and Y-ECM-gel significantly increased, and the effect was most evident in the Y-ECM-gel group (Figure 6C). Statistical quantitative analysis further confirmed this trend (Figure 6D). This indicates that Y-ECM-gel can effectively stimulate endogenous osteoblast lineages to leave their niche and migrate to bone defect sites. This effect may be due to the bioactive molecules present in Y-ECM-gel that promote recruitment.

Next, we evaluated whether Y-ECM-gel could promote MC3T3-E1 osteogenesis. As shown in Figure 6E, MC3T3-E1 co-cultured with the two hydrogels were stained with ALP and ARS, respectively, to evaluate early and late osteogenesis. Results showed that after 7 days of co-culture, ALP expression levels in the ECM-gel group and Y-ECM-gel group were significantly higher than in the control group, and Y-ECM-gel showed the strongest effect, indicating that Y-ECM-gel possesses the ability to promote early bone regeneration. Meanwhile, the number of ARS-stained calcific nodules was in line with the ALP staining trend, which indicated that Y-ECM-gel could promote late osteogenesis. ALP activity assays and ARS quantitative analysis also confirmed the same trend, and there was a statistically significant difference (Figure 6F). To further investigate osteogenesis at the molecular level, we analyzed the expression of osteogenesis-related genes and proteins using qRT-PCR and IF staining. As shown in Figure 6G, the expression levels of osteogenesis-related genes (ALP, BMP2, OCN, Runx2 and Col I) in the ECM-gel group and Y-ECM-gel group were significantly higher than those in the control group, with the Y-ECM-gel group exhibiting the most remarkable effect. Similar to the above findings, IF staining results (Figure 6H and I) revealed that compared to the control group, both ECM-gel and Y-ECM-gel significantly enhanced the expression of osteogenesis-related proteins (OCN and BMP2), with Y-ECM-gel showing the most remarkable effect. OCN is one of the most abundant noncollagenous proteins secreted by mature osteoblasts in the bone matrix [40]. The expression of OCN is commonly regarded as a marker of osteogenesis and maturation. BMP2 also plays a crucial role in the process of osteogenesis [41]. Quantitative statistical analysis revealed that the Y-ECM-gel group exhibited the highest expression levels of OCN and BMP2, and they were statistically significant. This further demonstrated the significant role of Y-ECM-gel in promoting MC3T3-E1 osteogenesis (Figure 6J). These results confirm that Y-ECM-gel can positively promote osteogenesis *in vitro*.

### Y-ECM-gel regulates the fate of MC3T3-E1 *in vitro*

**Figure 7:**
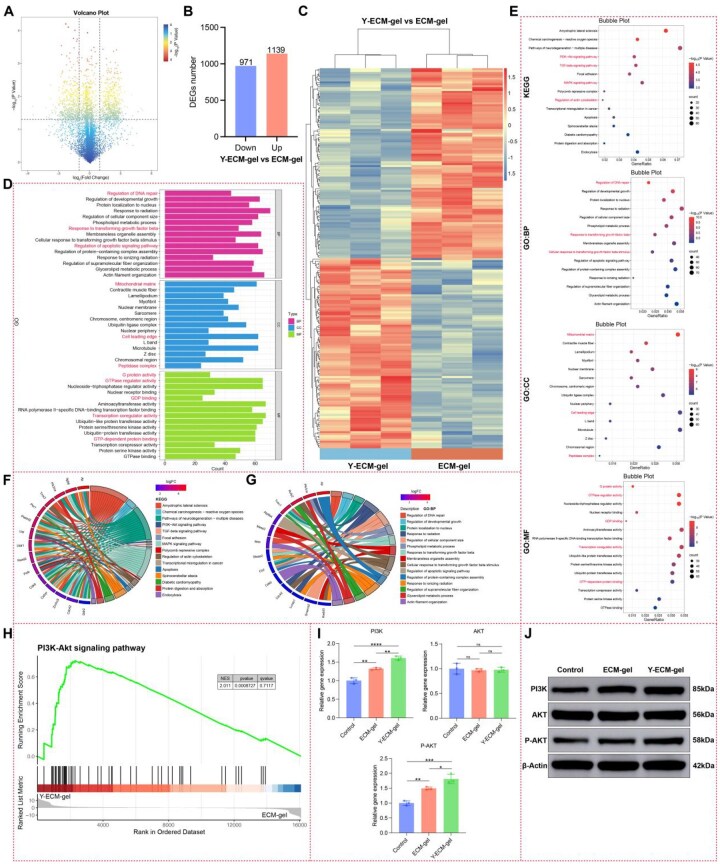
Analyses of RNA-seq and DEGs in MC3T3-E1 co-cultured with ECM-gel or Y-ECM-gel. (**A**) Volcano plot exhibiting the up or downregulation DEGs. (**B**) Numbers of DEGs in the two groups. (**C**) Heatmap analysis indicated 100 DEGs in the two groups. (**D**) GO analysis of the DEGs. The *x* and *y* axes represent the number of genes and GO terms. BP, CC and MF represent biological processes, cellular component and molecular function. (**E**) Bubble charts of the KEGG, GO-BP, GO-CC and GO-MF enrichment analysis for DEGs. The *x* and *y* axes represent the GeneRatio and KEGG terms, respectively. (**F**) Enriched chord diagram of the KEGG analysis. (**G**) Enriched chord diagram of the GO-BP analysis. (**H**) GSEA and standardized enrichment score (NES). (**I**) qRT-PCR analysis of gene expression levels of PI3K/AKT signaling pathway-related genes (PI3K, AKT and P-AKT). (**J**) Western blot analysis of protein expression levels of PI3K/AKT signaling pathway-related proteins (PI3K, AKT and P-AKT). Error bars denote means ± SD, *P < 0.05, **P < 0.01, ***P < 0.001, ****P < 0.0001, ns indicates no significance.

An illuminating exploration of the mechanisms that steer MC3T3-E1 fate was undertaken through an RNA-seq analysis of cells co-cultured with Y-ECM-gel. Principal component analysis (PCA) revealed a clear difference in all genes between ECM-gel group and Y-ECM-gel group, which emphasized the differences in their transcriptional profiles (Supplementary Figure S7). The study identified a total of 2110 DEGs, comprising 971 downregulated genes and 1139 upregulated genes. The screening criteria were adj. *P* values < 0.05 and |log2(Fold Change)| > 1 (Figure 7A and B). The heatmap lists the top 100 DEGs with the most significant changes (Figure 7C). We performed GO and KEGG enrichment analyses on DEGs. GO analysis subdivides DEGs into BP, CC and MF. Barplot and bubble plot present specific terms enriched in DEGs in different styles (Figure 7D and E). For BP, DEGs were primarily enriched in the terms of regulation of DNA repair, regulation of developmental growth, response to transforming growth factor beta, regulation of apoptotic signaling pathway, and regulation of protein-containing complex assembly. At the CC level, DEGs are strongly associated with the mitochondrial matrix, cell leading edge, chromosomal region and peptidase complex. In the MF category, DEGs were primarily enriched in G protein activity, GTPase regulator activity, GDP binding, transcription coregulator activity and GTP-dependent protein binding. The enriched terms described above are crucial for the process of bone regeneration. KEGG pathway analysis revealed (Figure 7E) that pathways associated with cell proliferation and differentiation, such as the PI3K/AKT signaling pathway, TGF-beta signaling pathway and MAPK signaling pathway, were significantly upregulated. Among these, the regulation of the actin cytoskeleton was also enriched. Based on GO-BP and KEGG analyses, along with enriched chordal diagrams (Figure 7F and G), Atr, Atp8b4, Pik3ca, Tmx3 and Cdc42 may serve as key regulators in relevant biological processes, which promote MC3T3-E1 proliferation and osteogenesis. Gene set enrichment analysis (GSEA) further verified the regulatory capacity of Y-ECM-gel in MC3T3-E1 osteogenesis, demonstrating its ability to upregulate osteogenesis-related pathways (PI3K/AKT signaling pathway) [42] (Figure 7H). In the KEGG results, the PI3K/AKT signaling pathway stands out in terms of significance and enrichment. The PI3K/AKT pathway is a critical intracellular hub during bone regeneration, widely involved in the survival, proliferation, osteogenic differentiation and mineralization of osteoblasts and MSCs. It is also closely linked to angiogenesis and bone remodeling [43]. In bone regeneration models, PI3K/AKT pathway has also been confirmed as an important pathway promoting bone healing. Dong *et al*. [44] found that PI3K inhibitors significantly inhibited fracture healing in a mouse fracture model. Activation of the PI3K/AKT signaling pathway enhances osteoblast proliferation, differentiation and mineralization, ultimately accelerating fracture repair. To further validate the differential expression of key genes and proteins in the PI3K/AKT signaling pathway following Y-ECM-gel treatment, qRT-PCR and WB analysis were employed. Results showed that Y-ECM-gel promoted PI3K expression and activated AKT phosphorylation (Figure 7I and J). In conclusion, these results indicate that Y-ECM-gel may positively regulate osteogenesis through mechanisms such as promoting cellular DNA repair, modulating active proteases and activating the PI3K/AKT signaling pathway.

### Y-ECM-gel promotes HUVECs angiogenesis *in vitro*

**Figure 8:**
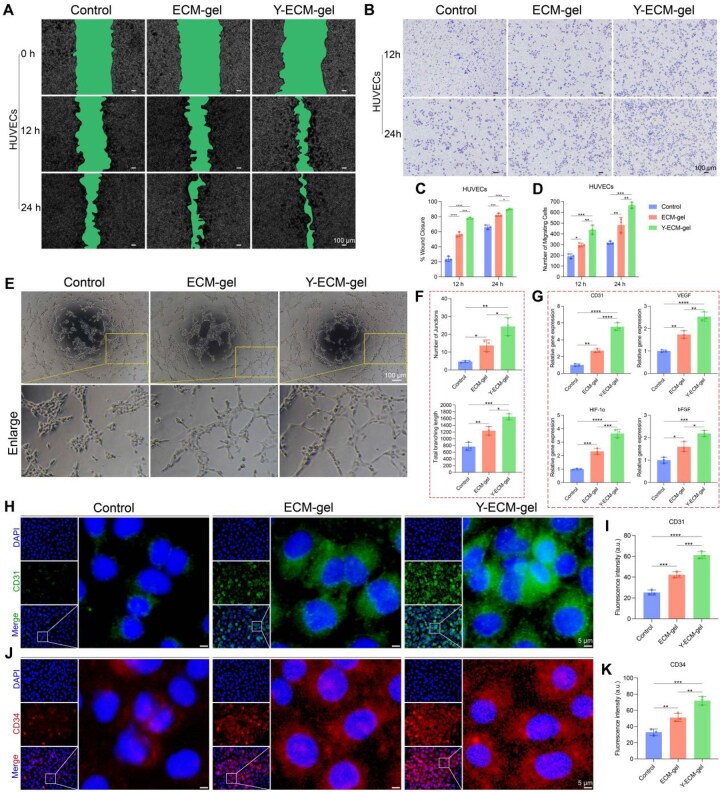
Y-ECM-gel promotes HUVECs angiogenesis *in vitro*. (**A**) Scratch assay verified the effect of Y-ECM-gel on promoting HUVECs migration at 0, 12 and 24 h. (**B**) Transwell assay accessing the migration activity of HUVECs cultured with the Y-ECM-gel where the migrated cells were stained with crystal violet. (**C**) Quantitative analysis of the wound closure (*n* = 3). (**D**) Quantitative analysis of the number of migrated HUVECs (*n* = 3). (**E**) Representative images of the tube formation assay. (**F**) Quantitative analysis of the tube formation structure (*n* = 3). (**G**) The angiogenesis-related genes (CD31, VEGF, HIF-1α and bFGF) expression of HUVECs on Day 7, respectively (*n* = 3). (**H**, **J**) Representative images of CD31 and CD34 IF staining of HUVECs. (**I**, **K**) Semi-quantitative analysis of fluorescence intensity (*n* = 3). Error bars denote means ± SD, *P < 0.05, **P < 0.01, ***P < 0.001, ****P < 0.0001.

As angiogenesis provides essential nutrients and growth factors for tissue during bone defect repair, bone regeneration is closely associated with angiogenesis [45–47]. Firstly, we examined the effect of Y-ECM-gel on HUVECs migration. Scratch assay results demonstrated that HUVECs in both the ECM-gel group and the Y-ECM-gel group exhibited significant wound-healing capacity at 12 h and 24 h, with the Y-ECM-gel group showing the strongest healing ability (Figure 8A). Statistical analysis also revealed significant differences (Figure 8C). Under the same conditions, the number of HUVECs migrating through the transwell chamber significantly increased in both ECM-gel group and Y-ECM-gel group, whether after 12 h or 24 h, with the highest number of migrated HUVECs in Y-ECM-gel group (Figure 8B). Quantitative analysis confirmed this trend (Figure 8D). Additionally, treatment with ECM-gel and Y-ECM-gel significantly promoted tube formation (Figure 8E), with Y-ECM-gel exhibiting the most notable effect. Quantitative analysis revealed that the Y-ECM-gel group exhibited the highest number of junctions and the greatest total branch length (Figure 8F). In addition, we detected the expression levels of angiogenesis-related genes (CD31, VEGF, HIF-1α and BFGF) via qRT-PCR. The results revealed that the expression levels of angiogenesis-related genes were most significantly elevated in HUVECs treated with Y-ECM-gel (Figure 8G). IF analysis revealed that the Y-ECM-gel group exhibited the highest fluorescence intensity for CD31 and CD34 at Day 7. Significant fluorescence signals were also observed in ECM-gel group, whereas minimal signals were detected in the control group (Figure 8H and J). Quantitative analysis of CD31 and CD34 fluorescence intensity confirmed the same trend (Figure 8I and K). These findings indicate that Y-ECM-gel can promote angiogenesis *in vitro*, providing strong experimental evidence for subsequent tube formation *in vivo*.

### Y-ECM-gel promotes new bone formation *in vivo*

**Figure 9:**
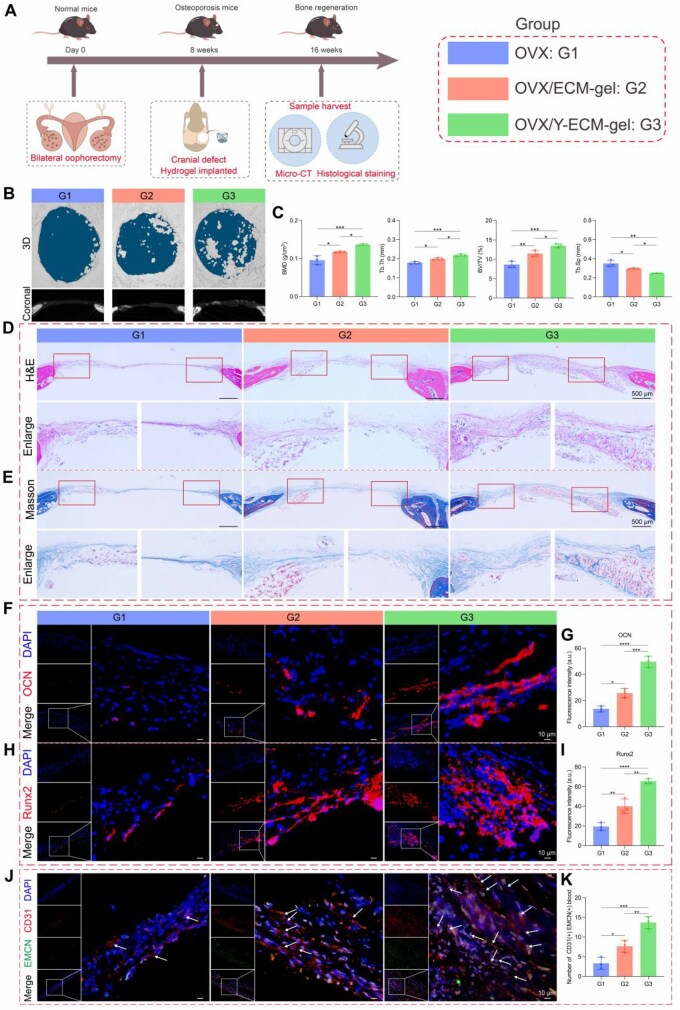
Y-ECM-gel promotes new bone formation *in vivo*. (**A**) Schematic illustration of the therapeutic procedure of the animal experiments. (**B**) Micro-CT images of the skulls of mice subjected to different treatments. (**C**) BMD, Tb.Th, BV/TV and Tb.Sp of the skulls in the different groups after treatment (*n* = 3). (**D**) Representative images of H&E staining of the bone defect region. (**E**) Representative images of Masson trichrome staining of the bone defect region. (**F**, **H**) Representative images of OCN and Runx2 IF staining. (**G**, **I**) Semi-quantitative analysis of OCN and Runx2 fluorescence intensity (*n* = 3). (**J**) Representative images of CD31 and EMCN IF co-localization staining. (**K**) Quantitative analysis of CD31 and EMCN double-positive number (*n* = 3). Error bars denote means ± SD, *P < 0.05, **P < 0.01, ***P < 0.001, ****P < 0.0001.

To evaluate the bone regenerative capacity of our engineered ECM hydrogel in osteoporotic bone defects, a stable OVX model was established in C57BL/6 mice via bilateral ovariectomy (Figure 9A). Six weeks postsurgery, we collected and analyzed uterine tissue. Body weight was measured weekly, revealing that ovariectomy resulted in weight loss (Supplementary Figure S8A). By Week 6, the uterine weight in the OVX group was significantly lower than that in the sham group (Supplementary Figure S8B).

Following the establishment of an OVX mice model, surgical procedures were performed in each group to create cranial defects (4 mm diameter). ECM-gel and Y-ECM-gel were implanted into the defect sites, respectively (Supplementary Figure S9). The groups included G1 as the control group (OVX and bone defect without any treatment), G2-G3 (OVX and bone defect model) mice, which received a local treatment in which ECM-gel or Y-ECM-gel were implanted into the defect sites, respectively. Eight weeks postoperatively, Micro-CT was employed to reconstruct the cranial defects and evaluate new bone formation. As shown in Figure 9B, 3D reconstructions, sagittal and coronal images reveal that both G2 and G3 exhibit more new bone formation compared to G1. Worth mentioning, the G3 shows a larger area of bone defect filled with new bone tissue, demonstrating more excellent regenerative performance. Additionally, bone volumetric analysis results—including BMD, Tb.Th, BV/TV and Tb.Sp—further confirm that both ECM-gel and Y-ECM-gel significantly promote bone regeneration even under osteoporotic conditions, with Y-ECM-gel exhibiting the most notable osteogenic effect (Figure 9C). To summarize, micro-CT results demonstrate that our designed Y-ECM-gel significantly enhances bone repair performance even under osteoporotic conditions.

Additionally, histological staining was performed to evaluate new bone formation around the cranial defect at 8 weeks postoperatively. H&E staining revealed minimal new bone tissue at the margins of the cranial defect in G1, indicating poor bone repair capacity under osteoporotic conditions. In contrast, substantial new bone tissue was observed in the bone defect regions of both G2 and G3. G3 exhibited a higher degree of bone mineralization (Figure 9D). These findings demonstrate that Y-ECM-gel promotes osteogenesis and mineralization. Moreover, Masson’s trichrome staining results also revealed (Figure 9E) that the defect areas in G1 contained only sparse collagen fibers and fibrous tissue. In contrast, the defect areas in G2 and G3 were extensively covered by abundant collagen fibers and newly formed bone tissue. The results above demonstrate that both ECM-gel and Y-ECM-gel promote new bone formation. Meanwhile, Y-ECM-gel exhibits the optimal osteoporotic bone repair capability, in accordance with the expected outcome.

We further investigated the osteogenic and angiogenic effects of engineered ECM hydrogels in osteoporotic bone repair through IF analysis. As shown in (Figure 9F and H), the fluorescence intensities of OCN and Runx2 in G1 were the lowest among all groups, indicating reduced osteogenic activity in OVX mice. In contrast, the fluorescence intensities of OCN and Runx2 in G2 and G3 were significantly elevated. Among all groups, G3 exhibited the highest fluorescence intensity. Quantitative fluorescence analysis confirmed these findings with statistically significant differences (Figure 9G and I). Additionally, co-localization staining of CD31 and endmucin (EMCN) revealed that G3 exhibited significantly more CD31 and EMCN co-localized expression compared to other groups (Figure 9J). Quantitative analysis further validated that Y-ECM-gel promotes *in vivo* angiogenesis (Figure 9K). To clarify whether this effect is purely structural or biochemical, ELISA was performed. Results demonstrated that concentrations of BMP2 and VEGF in the Y-ECM-gel showed significant increases compared to the ECM-gel, with statistically significant differences. However, no significant statistical differences were observed in TGF-β (Supplementary Figure S10). This indicates that Yoda1 treatment enhances the loading of osteogenic and angiogenic growth factors. Combining proteomic and ELISA results, the “angiogenesis-osteogenesis coupling” effect of Y-ECM-gel may be biochemical, though structural influences cannot be entirely ruled out. Further investigation is required. The results above indicate that Y-ECM-gel significantly enhances bone regeneration in osteoporotic bone defects by promoting osteogenesis and angiogenesis coupling.

## Conclusion

In conclusion, we utilized Yoda1 to activate the Piezo1 channel in MSCs, enabling them to secrete a more functional ECM, thereby enhancing the potential for future strategic applications of the Piezo1 channel. Furthermore, the preparation of ECM into hydrogels has broadened its application scope. We have demonstrated that Y-ECM-gel exhibits good biocompatibility. Y-ECM-gel not only promotes the proliferation and migration of MC3T3-E1 but also facilitates their osteogenesis by activating the PI3K/AKT signaling pathway. Y-ECM-gel also promotes angiogenesis. In the osteoporotic bone defect model, Y-ECM-gel significantly enhances bone regeneration in the defect area by coordinating osteogenic and angiogenic coupling. These results highlight the practicality of Y-ECM-gel as a novel therapeutic strategy for enhancing bone regeneration, offering a feasible alternative to traditional decellularized matrix and synthetic materials. The importance of this research extends beyond structural support, it offers a new perspective on the concept of cell-free grafts for bone reconstruction. Future research should focus on the development of tunable, intelligent ECM hydrogels, which can precisely regulate mechanical properties and degradation rates. Additionally, achieving spatiotemporal delivery of osteogenic-angiogenic signals (such as growth factors, exosomes or drugs) will be critical for synergistically regulating the osteogenesis-angiogenesis-immune microenvironment, thereby improving bone defect repair.

## Limitations

Although Y-ECM-gel has demonstrated excellent bone regeneration performance, several challenges remain. First, achieving reliable batch-to-batch consistency is still difficult during large-scale manufacturing. Second, while its short-term *in vivo* safety has been verified, further studies are required to evaluate its long-term safety. Third, Y-ECM-gel still contains trace amounts of DNA, which may pose a potential risk of immunogenicity and immune rejection. At last, because the cranium is a nonload-bearing bone, this model cannot fully recapitulate the mechanically regulated regenerative processes of load-bearing bones and cranial healing occurs predominantly through intramembranous ossification. Therefore, our current findings are insufficient to directly extrapolate its performance under physiological weight-bearing, including structural support, material retention and mechanosensitive remodeling. In future work, we will validate Y-ECM-gel in osteoporotic load-bearing bone defect models (e.g. femoral defects) and incorporate assessments of mechanical properties and functional integration.

## Supplementary Material

rbag080_Supplementary_Data

## Data Availability

Data will be made available on request.
