# Supplementary material for: Piezo1-activated mesenchymal stem cells-derived extracellular matrix hydrogel promotes the repair of osteoporotic bone defects through osteogenic and angiogenic coupling
Source: Regen Biomater. 2026 May 27;13:rbag080. doi: 10.1093/rb/rbag080 (PMC13250948; doi:10.1093/rb/rbag080)
Supplement: rbag080_Supplementary_Data [file rbag080_supplementary_data.docx]

**Supplementary Materials**

| 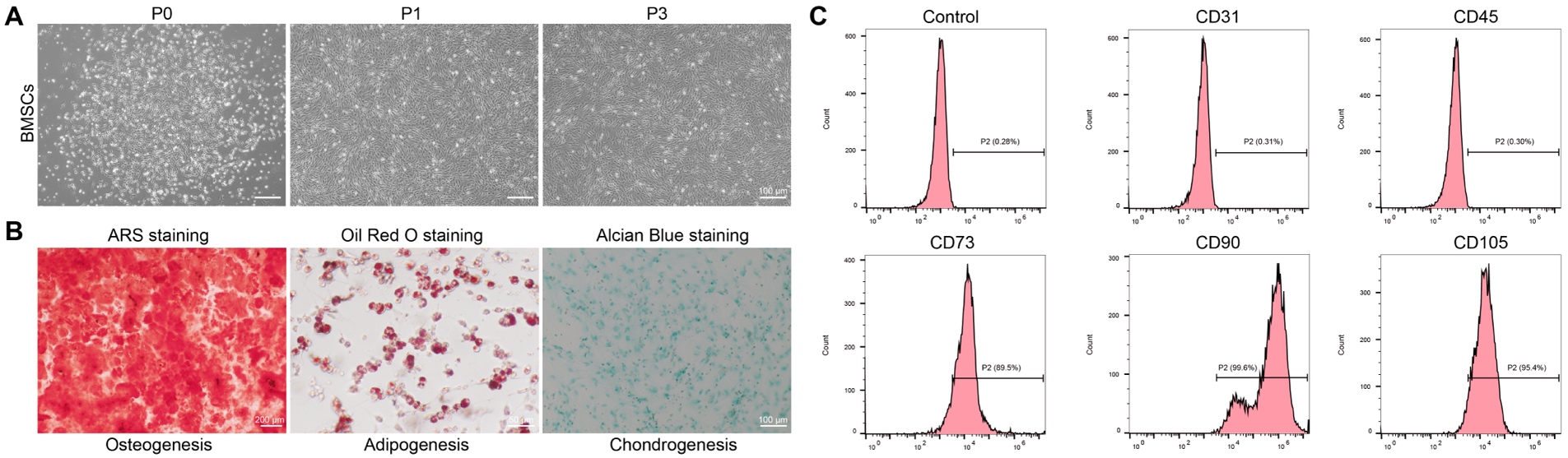 |
| --- |
| **Figure S1.** Isolation and characterization of BMSCs. (**A**) Morphology of P0, P1, and P3 BMSCs. (**B**) Three lineage differentiation of BMSCs (Osteogenesis, Adipogenesis, Chondrogenesis). (**C**) Identification of MSCs markers (CD31, CD45, CD73, CD90, and CD105) by flow cytometry. |
| 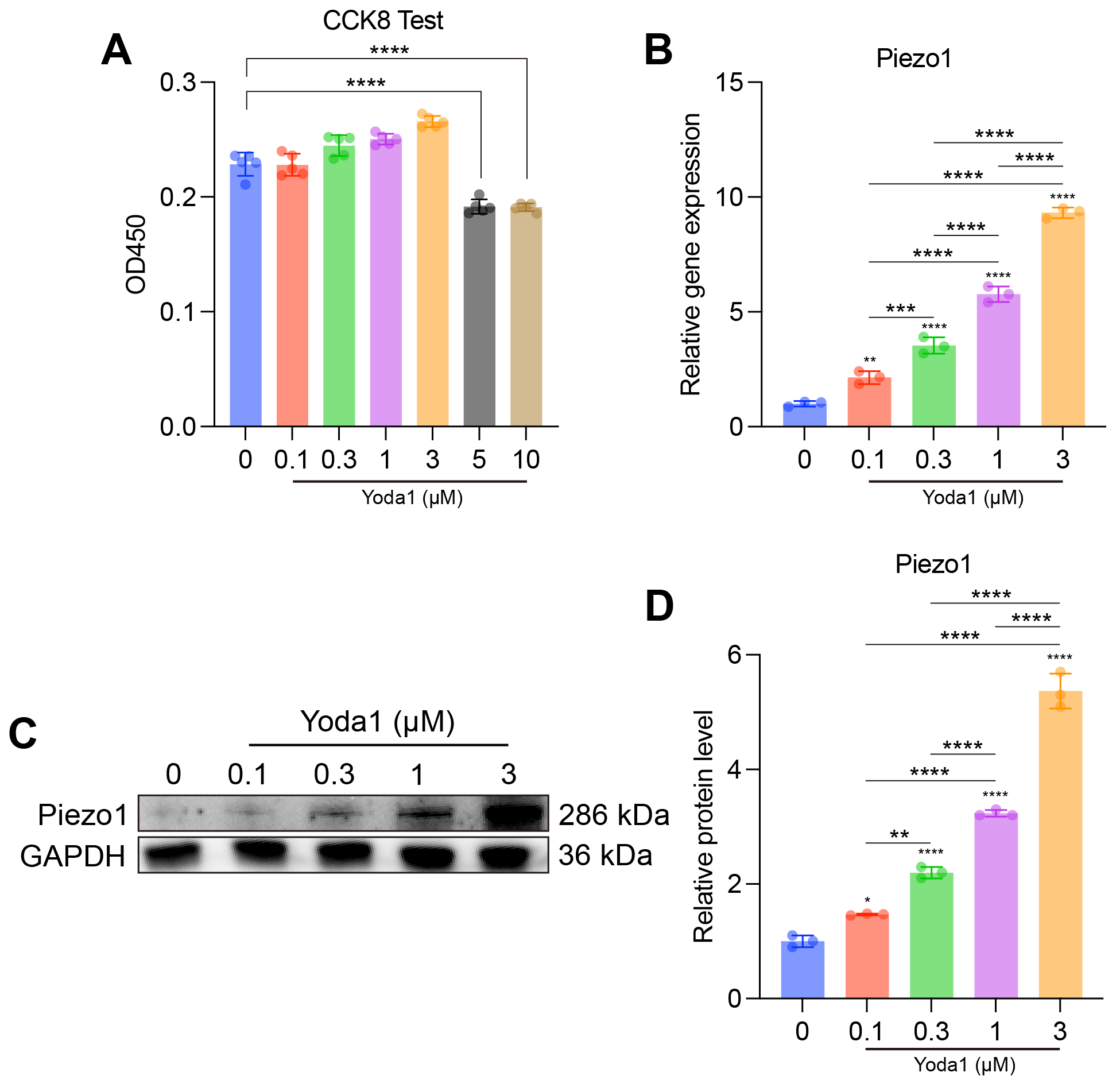 |
| **Figure S2.** Determination of Yoda1 concentration. (**A**) CCK-8 results of BMSCs viability under the treatment of Yoda1with different concentrations (n = 5). (**B**) qRT-PCR analysis of the expression of Piezo1 in BMSCs under the treatment of Yoda1with different concentrations (n = 3). (**C**) WB analysis of the expression of Piezo1 in BMSCs under the treatment of Yoda1with different concentrations (n = 3). (**D**) Quantitative analysis results of the WB experiment (n = 3). Error bars denote means ± SD, *P<0.05, **P<0.01, ***P<0.001, ****P<0.0001. |
| 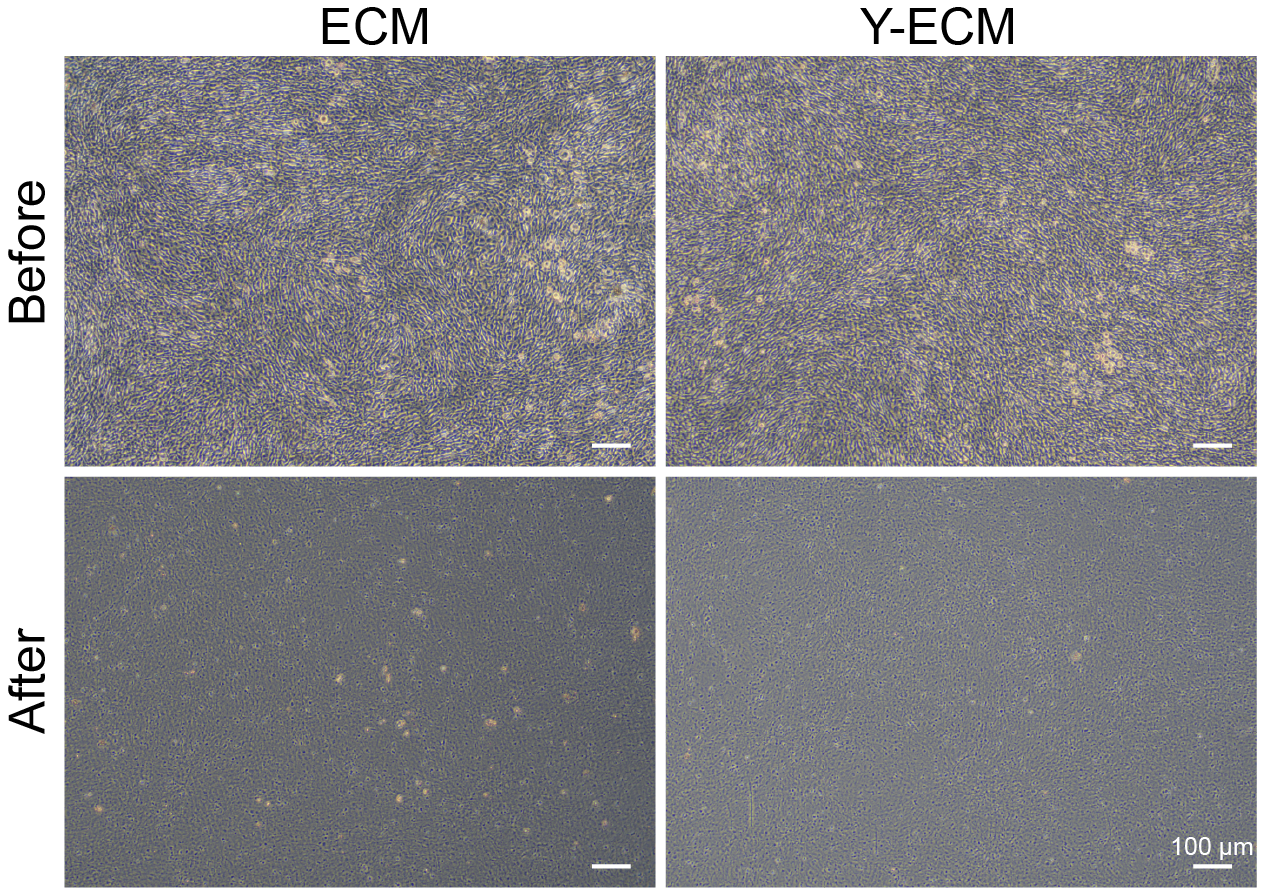 |
| **Figure S3.** Microscopic observations of BMSCs before and after decellularization. |
| 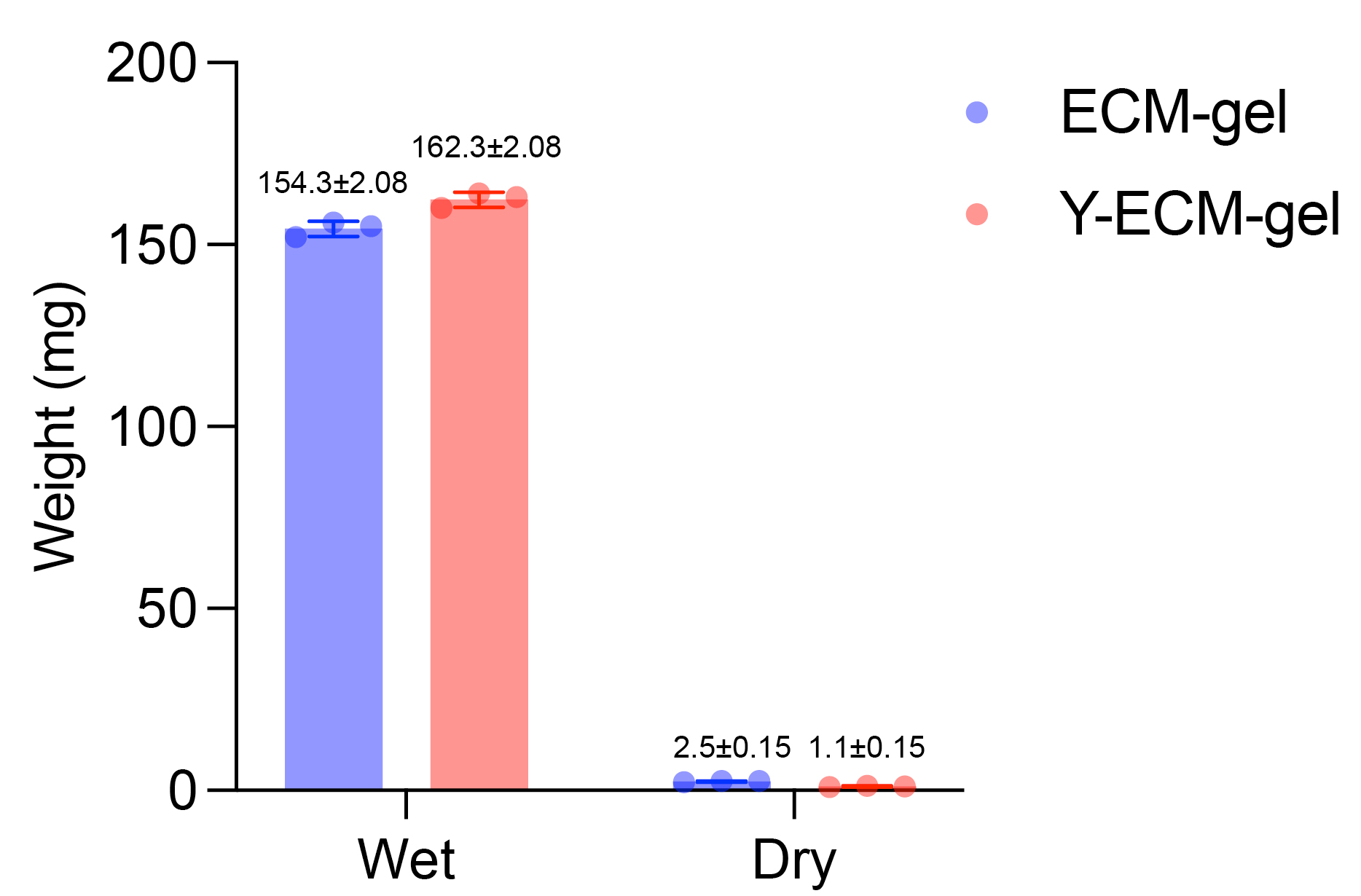 |
| **Figure S4.** Wet and dry weight of ECM-gel and Y-ECM-gel.  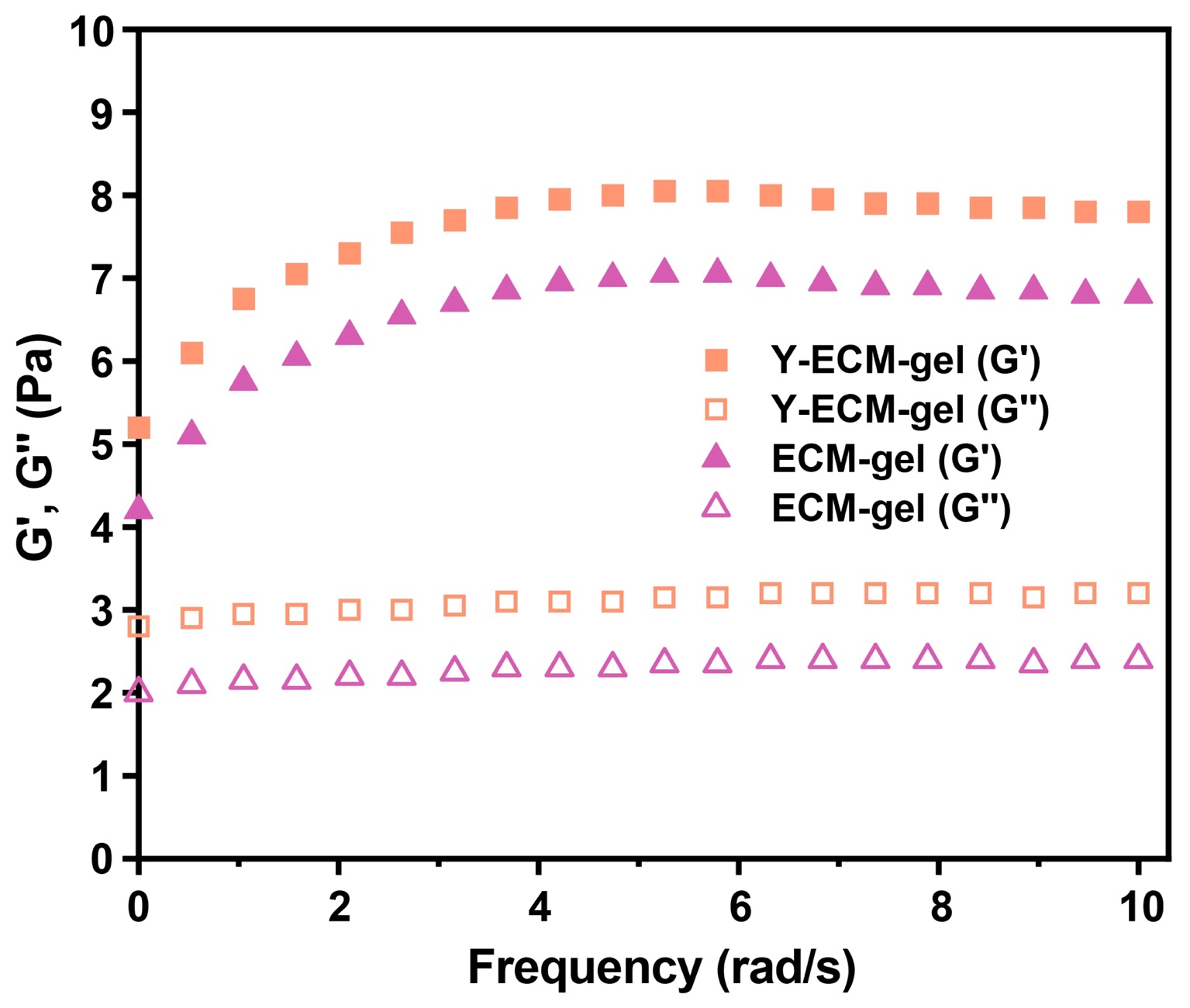  **Figure S5.** Rheological property of Y-ECM-gel and ECM-gel.  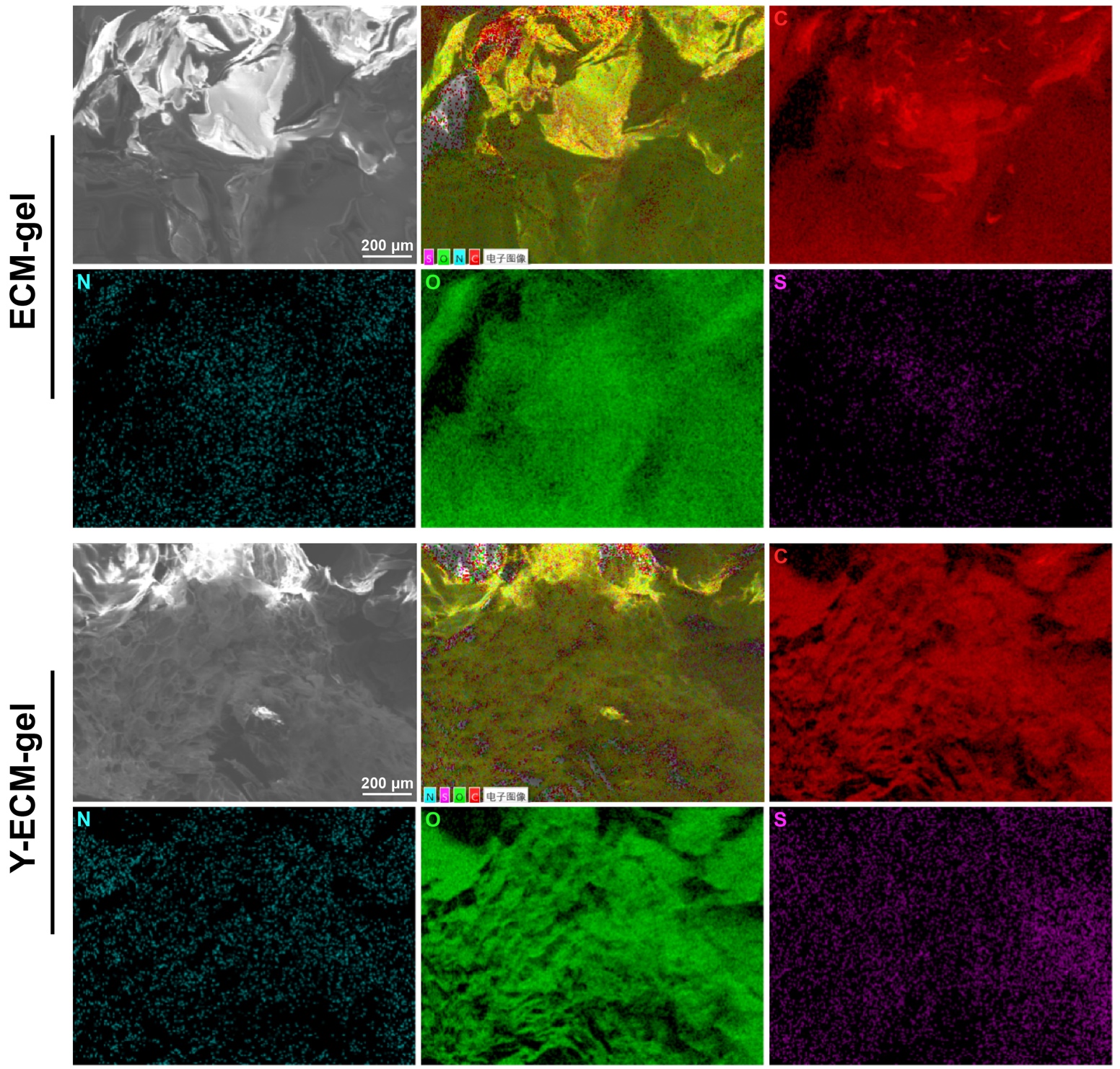 |
| **Figure S6.** Elemental mapping images of ECM-gel and Y-ECM-gel captured by SEM. carbon (C), nitrogen (N), oxygen (O), and sulfur (S). |
| 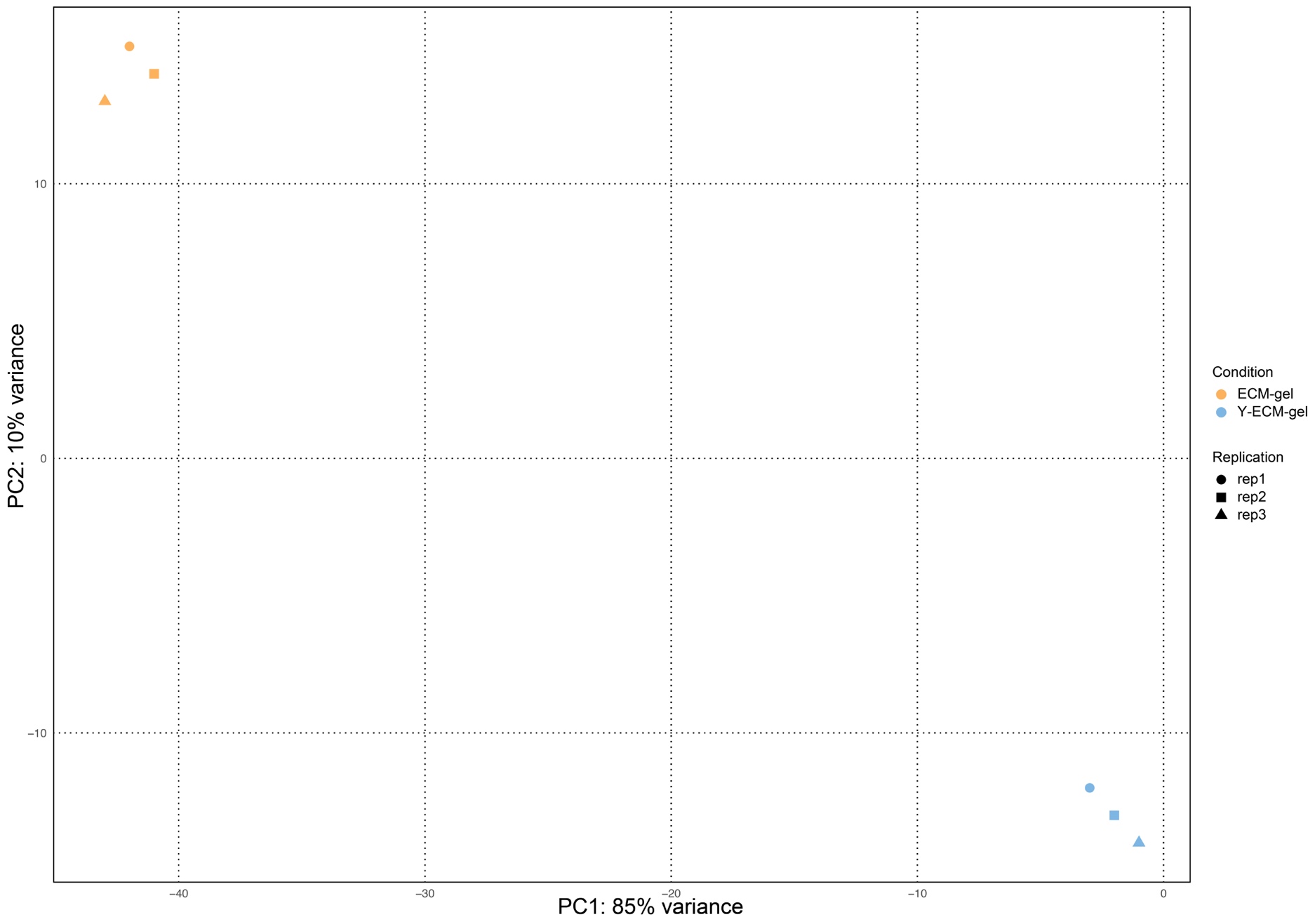 |
| **Figure S7.** PCA of DEGs in the two groups (n = 3). |
| 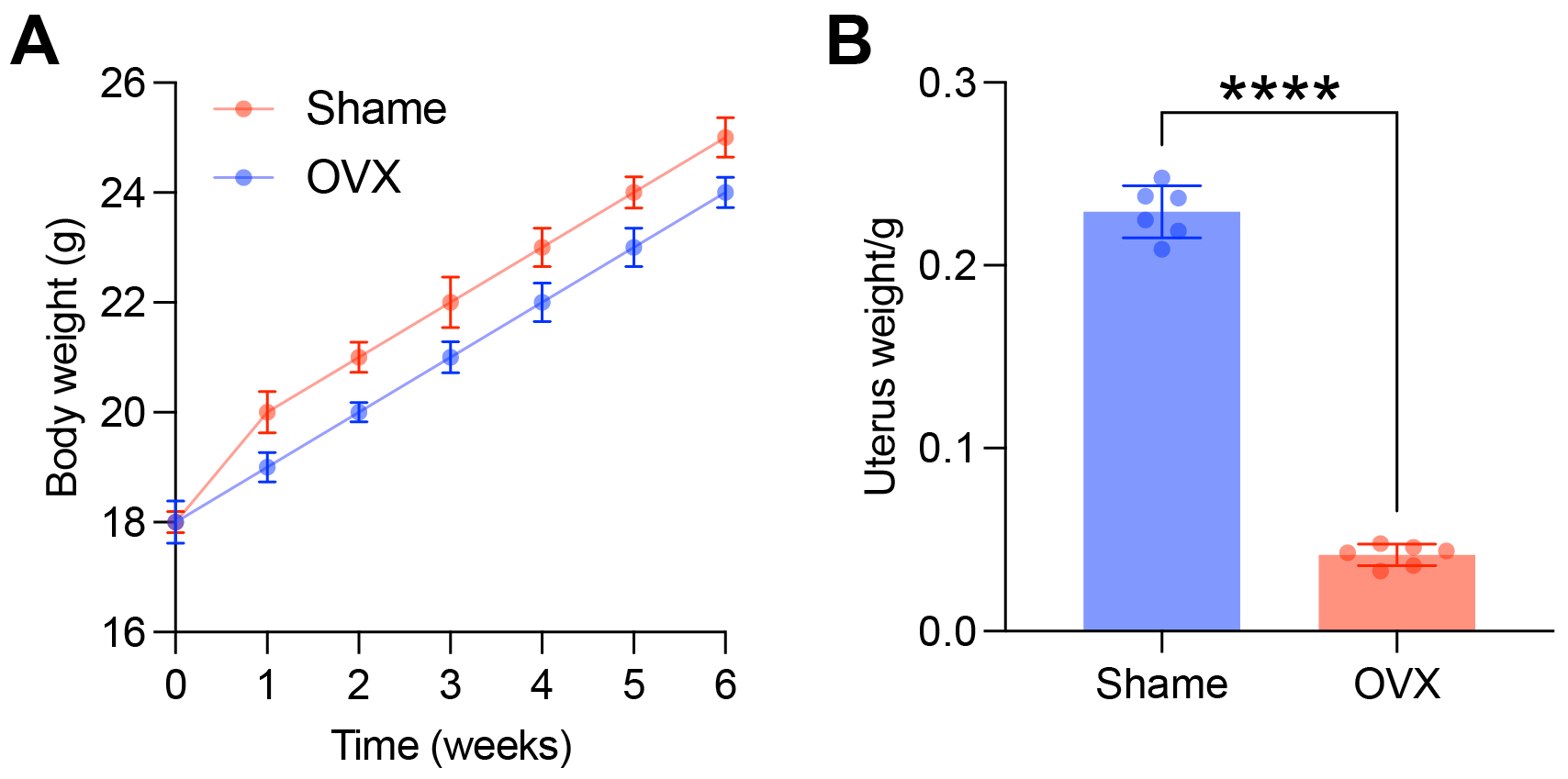 |
| **Figure S8.** Evaluation of the OVX Model. (**A**) Changes in body weight of mice after bilateral ovariectomy. (**B**) Weight of the uterus after bilateral oophorectomy. Error bars denote means ± SD, ****P<0.0001.  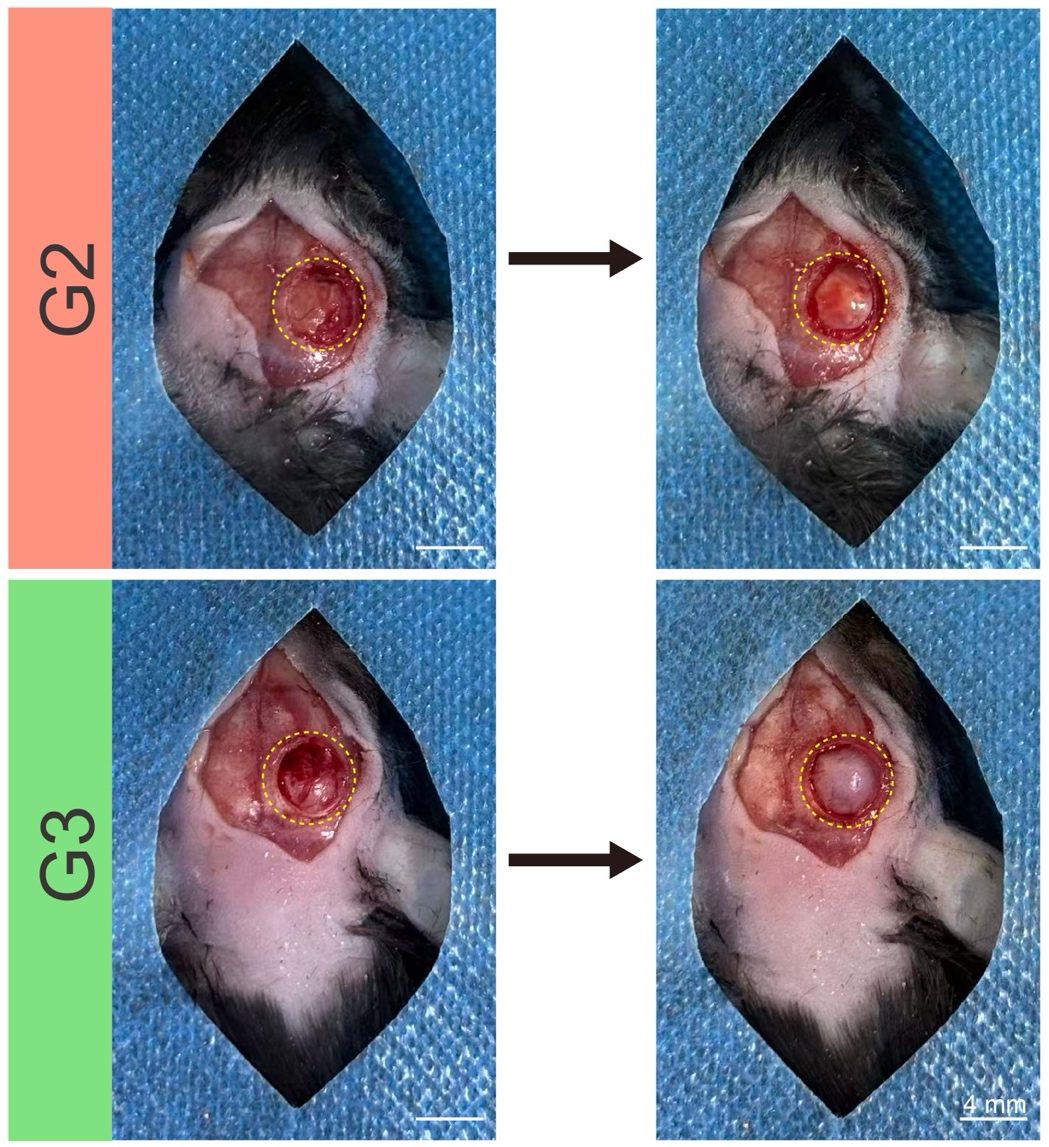  **Figure S9.** Cranial defect creation and hydrogel implantation. |


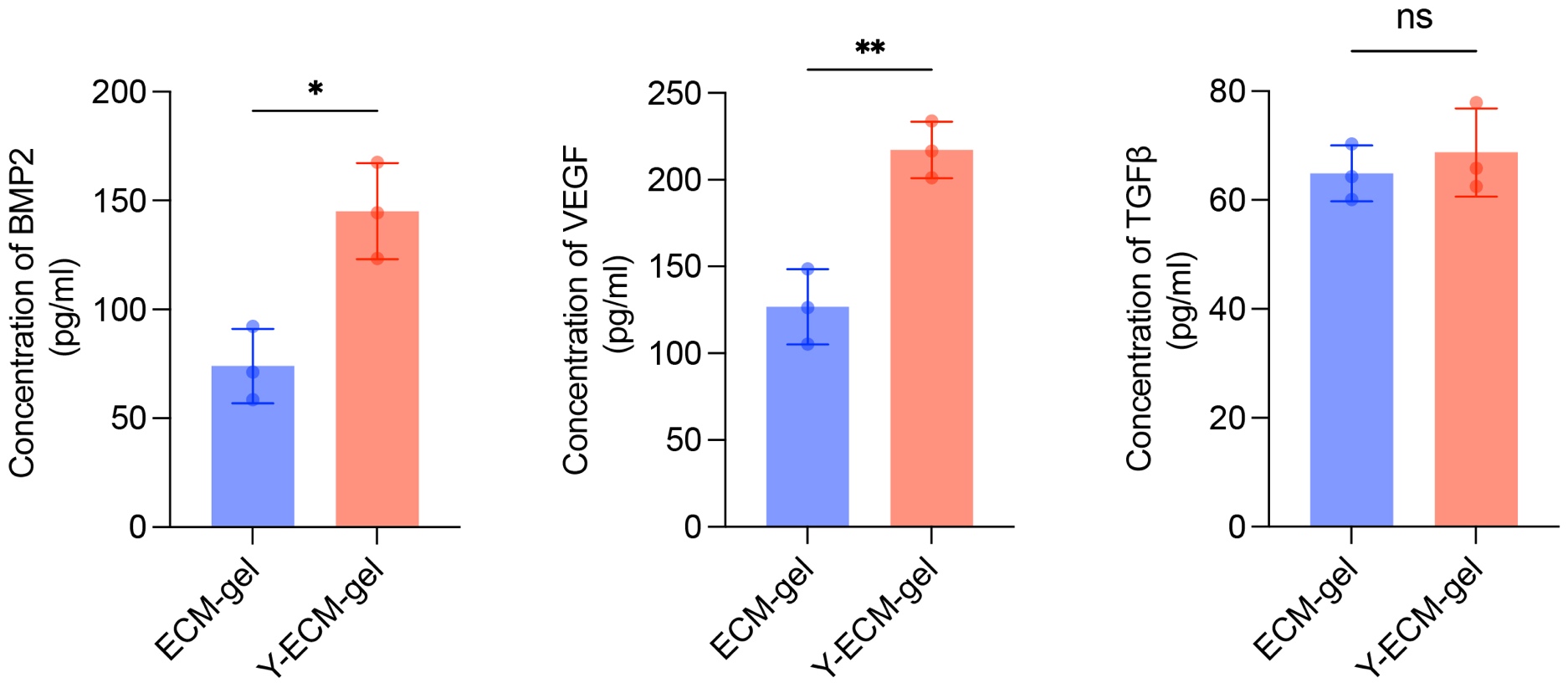


**Figure S10.** Concentration of BMP2, VEGF, and TGF-β in ECM-gel and Y-ECM-gel were quantified by ELISA.

**Table S1.** Primers for RT-PCR

| Gene | Forward premier | Reverse premier |
| --- | --- | --- |
| GAPDH | GCAAAGTGGAGATTGTTGCCAT | CCTTGACTGTGCCGTTGAATTT |
| Piezo1 | TCATCATCCTTAACCACATGGTG | TGAAGACGATAGCTGTCATCCA |
| ALP | CCAACTCTTTTGTGCCAGAGA | GGCTACATTGGTGTTGAGCTTTT |
| BMP2 | GGGACCCGCTGTCTTCTAGT | TCAACTCAAATTCGCTGAGGAC |
| OCN | GCAGCTTGGTGCACACCTAG | GGAGCTGCTGTGACATCCAT |
| Runx2 | GACTGTGGTTACCGTCATGGC | ACTTGGTTTTTCATAACAGCGGA |
| Col 1 | GCTCCTCTTAGGGGCCACT | CCACGTCTCACCATTGGGG |
| CD31 | ACCGTGACGGAATCCTTCTCT | GCTGGACTCCACTTTGCAC |
| VEGF | CCACGTCAGAGAGCAACATCA | TCATTCTCTCTATGTGCTGGCTTT |
| HIF-1α | GGGAGATCTGGGGACAGGAGGATCGCC | GGGAAGCTCATAAAAAACTTTAGATTC |
| BFGF | CTCACGTGGCACCAGTGGAT | CACAGAGGATGAATAGTAGC |

# **Abbreviations**

| ALP | Alkaline phosphatase |
| --- | --- |
| ANOVA | One-way analysis of variance |
| ARS | Alizarin Red S |
| BMD | Bone mineral density |
| BMP2 | Bone morphogenetic protein 2 |
| BMSCs | Bone marrow mesenchymal stem cells |
| BP | Biological process |
| BV/TV | Bone volume fraction |
| CC | Cellular component |
| 3D | Three-dimensional |
| DEGs | Differentially expressed genes |
| DEPs | Differentially expressed proteins |
| ECM | Extracellular matrix |
| ECM-gel | MSCs-derived ECM hydrogel |
| ELISA | Enzyme-linked immunosorbent assay |
| EMCN | Endmucin |
| FBS | Fetal bovine serum |
| FDR | False discovery rate |
| FTIR | Fourier transform infrared spectroscopy |
| GAGs | Glycosaminoglycans |
| GO | Gene Ontology |
| H&E | Hematoxylin and eosin |
| HUVECs | Human umbilical vein endothelial cells |
| IF | Immunofluorescence |
| KEGG | Kyoto Encyclopedia of Genes and Genomes |
| MC3T3-E1 | Preosteoblast cells |
| MF | Molecular function |
| MSCs | Mesenchymal stem cells |
| OCN | Osteocalcin |
| OP | Osteoporosis |
| OVX | Ovariectomized |
| PCA | Principal component analysis |
| PSM | Peptide spectrum matching |
| qRT-PCR | Quantitative real-time polymerase chain reaction |
| Runx2 | Runt-related transcription factor 2 |
| SD | Standard deviation |
| SEM | Scanning electron microscopy |
| Tb.Sp | Trabecular separation |
| Tb.Th | Trabecular thickness |
| TCP | Tissue culture plates |
| Y-ECM | Yoda1-treated MSCs-derived ECM |
| Y-ECM-gel | Piezo1-activated MSCs-derived ECM hydrogel |
